# Preschool environment and preschool teacher’s physical activity and their association with children’s activity levels at preschool

**DOI:** 10.1371/journal.pone.0239838

**Published:** 2020-10-15

**Authors:** Chu Chen, Viktor H. Ahlqvist, Pontus Henriksson, Cecilia Magnusson, Daniel Berglind

**Affiliations:** 1 Centre for Epidemiology and Community Medicine, Region Stockholm, Stockholm, Sweden; 2 Department of Global Public Health, Karolinska Institutet, Solna, Sweden; 3 Department of Health, Medicine and Caring Sciences, Linköping University, Linköping, Sweden; University of Idaho, UNITED STATES

## Abstract

**Objective:**

The aim of this study was to investigate the association between preschool playground size, formalized physical activity (PA) policies, time spent outdoors and preschool teacher’s levels of PA and children’s objectively assessed levels of PA and sedentary time (ST) during preschool hours.

**Methods:**

In total, 369 children and 84 preschool teachers from 27 preschools in Södermalm municipally, Stockholm Sweden wore an Actigraph GT3X+ accelerometer during 7 consecutive days. Preschool environmental and structural characteristics were measured via the Environment and Policy Evaluation Self-Report (EPAO-SR) instrument and time in- and outdoors was recorded by preschool teachers during the PA measurements. Weight and height of children were measured via validated scales and parents filled out a questionnaire on demographical and descriptive variables. Linear mixed models, nested on preschool level, were used to assess the association between predictors and outcomes.

**Results:**

The mean child age was 4.7 years (SD 0.8) and 45% were girls. We found that children were more active in preschools with a formalized PA policy, compared to preschools without such a policy, but not less sedentary. The association between policy and activity seemed to be more pronounced when accounting for other environmental factors. Similar associations were found in children spent most time outdoors (uppermost quartile) compared with children spent least time outdoors (Lowermost quartile). Preschool teachers’ light PA (LPA) (*ß* = 0.25, *P* = 0.004) and steps (*ß* = 0.52, *P*<0.001) were associated with children’s LPA and steps while the preschool playground size showed no association with PA in children, when accounting for other environmental factors.

**Conclusion:**

The current study showed that preschool structural characteristics such as formalized PA policies and more time spent outdoors were positively associated with children’s PA. These findings suggest that formalized PA policies and time outdoors may be of importance for promoting children’s PA during preschool hours.

## Introduction

Total physical activity (PA), moderate to vigorous PA (MVPA) [1] and steps per day [2] are positively associated with multiple health indicators in young children, while more conflicting findings have been reported for sedentary time (ST) [3]. Furthermore, several studies have shown that physically active children tend to remain more physically active across their lifespan [4]. Despite the many known benefits of PA, children are in general physically inactive [5]. A review on preschoolers’ physical activity level based on objective measure has shown highly variable results that preschool children spend 2%–41% of their day in MVPA, 4%–33% in light PA (LPA), and 34%–94% sedentary [6]. Moreover, Swedish data with objectively measured PA and ST, show that preschoolers’ levels of PA are low [7, 8]. Hence, effective strategies informed by objective data are urgently required to promote child PA.

A theory base is suggested to be beneficial for the effectiveness of PA promoting strategies [9], therefore the social ecological model was employed as framework to understand systematically what factors may enable effective PA promotion in preschool children [10]. According to the social ecological model, there are different levels of determinants of health behaviors such as physical activity namely individual level, interpersonal level, organizational level and physical environment level [10]. Environmental intervention in preschool which lay emphasize on non-individual level determinants of PA, can potentially be an effective PA promotion strategy also addressing sustainability and equity but the evidence-base is scarce [11]. In Sweden, approximately 92% of all children 1–5 years of age are attending preschools, regardless of their parents’ socioeconomic status [12]. Furthermore, approximately 50% of children’s daily MVPA is accumulated during preschool hours [8]. Therefore, the preschool environment presents an ideal arena to promote early development of healthy PA and ST behaviors [13].

Potential modifiable characteristics for preschool includes physical environment, policy, time spent outdoors on the organizational level and teachers’ PA on the interpersonal level, but the evidence supporting effectiveness is preliminary. In terms of interventions to modify physical environment, studies have shown that structural environmental factors such as playground size, play equipment accessibility and design of the preschool playground may be of importance for children’s PA during preschool hours [14, 15]. However, consensus on playground size is hampered by the lack of evidence, application of objective measure on PA, and the difficulty in studying this issue with randomized experimental design [16]. Although physical environment level interventions address all children in the environment with potentially promising sustainability, they are seldom practical due to the requirement on resources especially on large scale [17].

Modifying organizational factors, such as policy and time spent outdoors, is less resource-dependent but may be effective provided adequate evidence base. Having a PA policy in preschool is suggested to be beneficial for preschooler’s PA but objective measure on PA is lacking [14, 18, 19]. Conflicting results have been demonstrated by the few existing studies with accelerometer data. While Dowda et al found more MVPA in PA promoting preschools where policy is one of the components [20], Erinosho et al showed a negative association between PA policy and accelerometer measured PA level in preschool children [21]. More research with objective PA data is needed to determine the association between policy and PA levels in preschool children. Similarly, studies investigating associations between time spent outdoors at preschool and children’s levels of PA using objective measures on PA are also scarce. Only a few existing studies indicate that the amount of time preschool children spend outdoors is positively associated with their levels of PA and negatively associated with ST [22, 23]. A recent randomized controlled trial showed that scheduling both shorter more frequent and longer outdoor sessions during preschool hours significantly increased preschool children’s MVPA [24]. Thus, increasing time spent outdoors during preschool hours may, in addition to policies and environmental factors, be an effective strategy to promote healthy PA among preschool children. However, more studies are warranted to further explore the potential of organizational level factors.

Interpersonal level factor such as preschool teacher’s attitude, initiative, and participation in physical activities along with children, may play an important role in promoting preschool children’s PA [25]. However, there is a lack of studies among the preschool population. Only one Norwegian study has used objectively measured PA in both preschool teachers’ and children and found a small, but statistically significant association, between preschool teachers’ and preschool children’s levels of PA during preschool hours [26]. More studies with objective measure on both preschool teachers and children are imperative to confirm the potential association between preschooler’s PA and preschool teachers’ PA.

To address these knowledge gaps, the aim of the current study is to assess to what extent the physical preschool environment, formalized PA policies, time spent outdoors and preschool teachers’ levels of PA were associated with children’s objectively assessed PA, steps and ST during preschool hours to deepen knowledge in informing strategy development for child PA promotion.

## Materials and methods

### Study design, setting and study population

In this cross-sectional observational study, 30 out of the total 51 municipal preschools within the Södermalm district of Stockholm Sweden, were invited to participate. Preschools were chosen to reflect a representative sample of the different environmental characteristics (outdoor operation and different size of the playground) within the Södermalm district. In Sweden, all children from the age of 1 to 5 are eligible to go to preschool. However, children aged 1–2 years are often separated in physical activity daily routines from children aged 3–5 years because of the difference in their development stage [27]. Further, WHO have formulated different physical activity guidelines due to this variation in growth between 1–2 years old toddlers and 3–5 years old preschool children [28]. As such, preschool children of 3–5 years old were chosen as the study population of this research and children between 3–5 years of age, at the participating preschools, were invited to participate. Written informed consent was obtained from all participating children’s parents and preschool teachers and the study has been approved by the Stockholm Ethical Review Board (EPN), Dnr: 2018/890-31/2. The fieldwork measurements, comprising questionnaires for preschool teachers and parents, body measures of children and 7 days of accelerometer measures of PA in children and preschool teachers, were carried out at the participating preschools from September to November 2018.

### Preschool environmental characteristics, policies and time outdoors

The Environment and Policy Evaluation Self-Report (EPAO-SR) Instrument, showing good to excellent validity and reliability [29], was administered to preschool teachers. The EPAO-SR instrument includes both questions about nutrition and physical activity and only questions regarding physical activity were distributed in this study. Subscales of the EPAO-SR were used to measure environmental characteristics and formalized PA policies in the participating preschools. The specific questions asked were “How large is your preschool playground?” and “Does your preschool has written policy or any other written document about physical activity? Answers about playground size were categorized and modified to include the Swedish outdoor activity practice; (1) ≤200m^2^, (2) around 900m^2^, (3) >2700m^2^ and (4) outdoors activity (all time at the preschool is spent outdoors). Formalized PA policy was analyzed as a dichotomous variable (Yes/No), depending on if the preschool had any written policy concerning PA or not. Time in-and outdoors was aggregated from in-out report, in which preschool teachers recorded time spent “indoors” or “outdoors” in 30-minute periods for every child on all weekdays during the PA measurements. Time outdoors was thereafter converted into quartiles (Q1 <138min, Q2 138min≤ to <187.5min, Q3 187.5min≤ to <234min and Q4 ≥234min), where Q1 comprises those 25% of preschool children who spent the least time outdoors and Q4 comprises those 25% who spent the most time outdoors.

### Body measures

Weight and height of participating children were measured via validated scales and stadiometers, respectively (calibrated scale: VB2-200-EC, Vetek AB, Väddö, Sweden; portable stadiometer: Seca 213, Seca, Chino, CA, USA). Body mass index (BMI) was classified as normal, overweight or obese according to an international classification by Cole et al., correcting for age and sex [30].

### Physical activity and sedentary time

PA and ST were measured via the triaxial Actigraph GT3X+ accelerometer, which has been tested extensively for reliability and validity and is widely used in epidemiological pediatric research [31]. Wear protocol and analyzing techniques followed best practices and used the latest recommendations to increase accuracy [31]: children and preschool teachers were instructed to wear the accelerometer, at the right hip, all waking hours for 7 consecutive days. A sampling rate of 60 Hz was used and vector magnitude (V_m_) activity counts (V_m_ = √ (X^2^ + Y^2^ + Z^2^)) was analyzed. Accelerometer data were considered valid if the child wore the accelerometer for at least 3 days, 10 hours/day. Non-wear time was defined as 60 or more consecutive minutes with zero counts, allowing up to 2 min of interruptions with non-zero counts [31]. Steps were determined using the manufacturer’s step algorithm, using the normal filter. MVPA, LPA and ST were calculated based on cut-offs and epochs developed specifically for the GT3X+ accelerometer, using V_m_ activity counts, in 4-year-old children [32]. A 60-s epoch length was used in analysis according to the epoch setting in the validation study that developed these cut-offs [32]. ST was calculated as any minute of less than 820 counts per minute (cpm), LPA as 820–3907 cpm and MVPA as ≥3908 cpm. For preschool teachers, MVPA, LPA and ST were calculated based on cut-offs and epochs developed by Santos-Lozano et al. for the GT3X+ accelerometer, using V_m_ activity counts [33]. ST was calculated as any minute of less than 150 cpm, LPA as 150–3207 cpm and MVPA as ≥3208 cpm [33]. After the validation and classification of PA level for whole day PA accelerometer measure, the time-stamped accelerometer data was further matched with preschool time information to extract PA during preschool time due to the focus of the preschool factors in this study. In Sweden, preschool hours vary to fit parents’ working schedule, but most preschools are open from 7:00 to 19:00. The preschool arrival and departure time information for each child was documented by preschool teachers daily in the same in/out report that recorded children’s activity indoor or outdoor in 30 minutes periods from 7:00 to 19:00.

### Family characteristics

At baseline, parents filled out a questionnaire on demographical and descriptive variables on anthropometry (height and weight) and highest education level, categorized into elementary school, upper secondary school and university education.

### Teacher PA

Teachers’ PA outcomes were aggregated at preschool level by calculating the means of the respective PA outcomes of all teachers in each preschool. Every outcome was then categorized into high and low by the median. Cut-offs for teacher PA outcomes aggregated at preschool level were defined as: MVPA_low_<24.5 min, LPA_low_<310.4 min, Steps_low_<6656 steps, ST_low_≤184.5 min.

### Statistical analyses

Descriptive analyses included the distribution (mean and standard deviation (SD)) of various background characteristics and PA outcomes by preschool policy, playground size, time outdoors and teacher PA.

Next, we used Linear Mixed Models (LMM), nested on preschool level, to examine associations between existence of formalized PA policy, playground size, time spent outdoors and preschool teachers aggregated levels of MVPA, LPA, steps and ST with child levels of MVPA, LPA, steps and ST. We analyzed each association between the exposures and outcomes independently and all predictors jointly in both unadjusted and adjusted models. Adjustments, in all models presented, were made for age of the child, sex and BMI [34] that has been selected based on causal diagram [35]. There are no defined classrooms in Swedish preschools where all teachers take care and interact will all children in principle [36]. Although children can be divided into groups, these groups are not fixed, and most children participate in different group constellations [27]. Therefore, a 2-level nesting, children/teachers nested in preschools, was adopted in the LMM. In addition, we estimated the intra class correlation for each mixed model to determine the preschool-level cluster effect.

All statistical analysis was performed in software STATA version 16.0.

## Results

**Fig 1:**
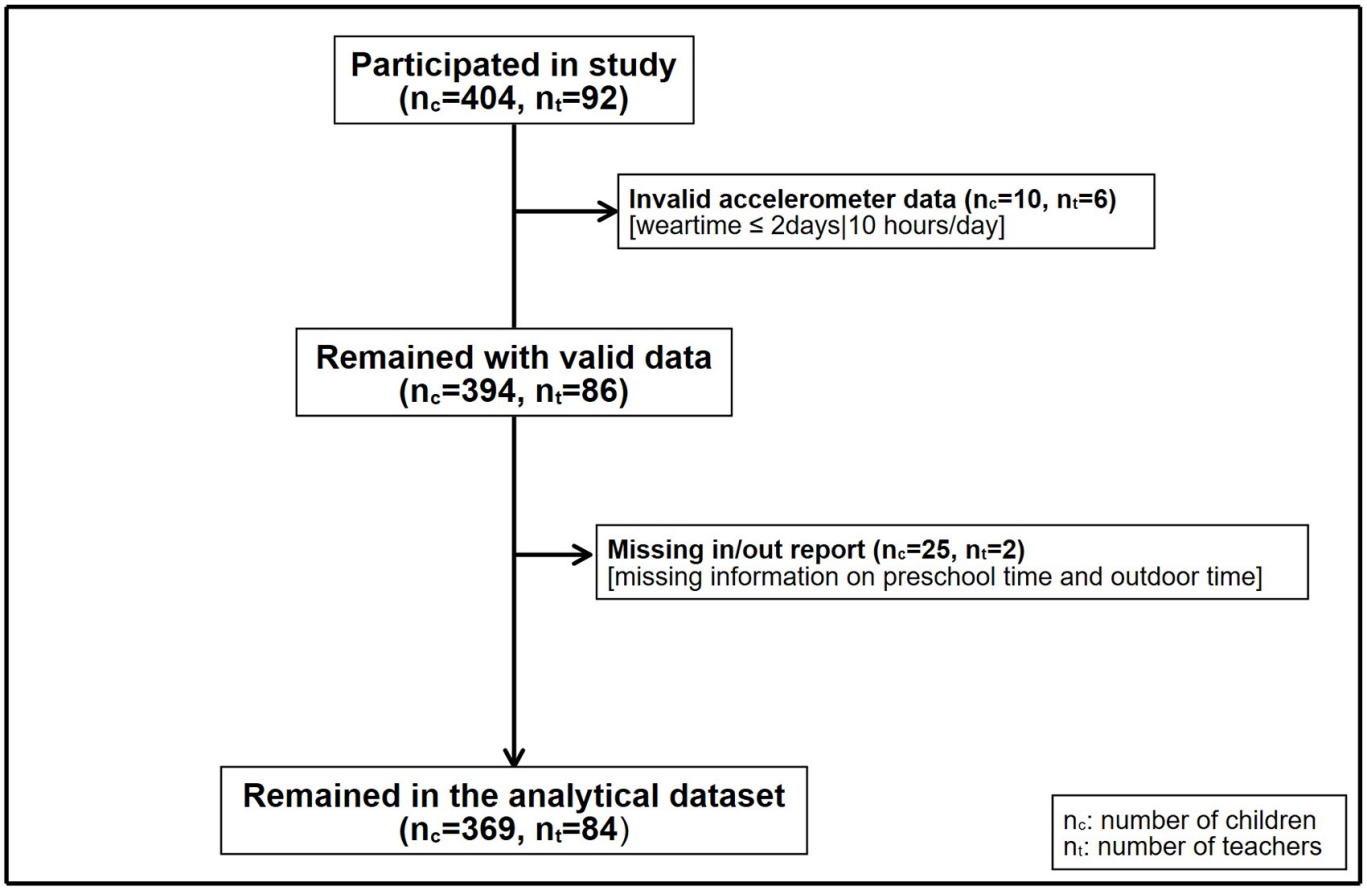
Flowchart of participants.

Fig 1 demonstrates the derivation of the analytical dataset. In total, 404 children and 92 preschool teachers from 27 preschools participated in the current study. First, 10 children and 6 teachers were excluded because they had less than 3 days or 10 hours/day of accelerometer data. Second, 25 children and 2 teachers were excluded due to missing recorded preschool hours, as such information were required to determine PA during preschool time. Thus, the final analytical sample comprised 369 children and 84 preschool teachers. The mean child age was 4.7 years (SD 0.1) and 45% were girls. On average, a child spent 475 minutes (7.9h) in preschool per day, of which 269 (SD 97.5) and 206 (SD 107.7) minutes were spent in- and outdoors, respectively.

Table 1 provides an overview of the preschool characteristics by preschool policy, playground size and time spent outdoors (exposures) and child’s daily average levels of PA, steps and ST (outcomes) during preschool hours. The overview of teacher’s PA, steps and ST aggregated on preschool level and child’s daily average of PA during preschool time, steps and ST respectively is presented in S1 Table. In total, girls spent 19% less minutes in MVPA, 5% less minutes in LPA and spend 14% more minutes in ST compared with boys during preschool hours.

**Table 1:** Descriptive characteristics in relation to preschool level features.

		Formalized PA Policy		Playground area (m^2^)				Time spent outdoors			
	Total	No	Yes	≤200	Around 900	>2700	Out group	Q1	Q2	Q3	Q4
	N = 369	N = 290	N = 79	N = 98	N = 69	N = 151	N = 51	N = 94	N = 93	N = 90	N = 92
Individual characteristics											
Boys, n (%)	204 (55.3)	125 (43.1)	40 (50.6)	55 (56.1)	34 (49.3)	88 (58.3)	27 (52.9)	40 (43)	50 (54)	38 (42)	37 (40)
Age, mean (SD)	4.7 (0.1)	4.6 (0.8)	4.9 (0.7)	4.7 (0.7)	4.6 (0.9)	4.7 (0.7)	4.4 (0.9)	4.8 (0.8)	4.6 (0.8)	4.7 (0.7)	4.5 (0.8)
BMI, mean (SD)	15.7 (0.1)	15.7 (2.8)	15.5 (2.1)	15.2 (3.8)	16.0 (1.2)	15.6 (2.5)	16.2 (1.7)	14.6 (4.3)	15.7 (2.0)	16.2 (1.1)	16.2 (1.6)
Overweight, n (%)	25 (6.8)	20 (6.9)	5 (6.3)	7 (7.1)	6 (8.7)	8 (5.3)	4 (7.8)	5 (5)	6 (6)	8 (9)	6 (7)
Obesity, n (%)	8 (2.2)	7 (2.4)	1 (1.3)	3 (3.1)	1 (1.4)	2 (1.3)	2 (3.9)	1 (1)	0 (0)	3 (3)	4 (4)
Preschool children’s physical activity level during preschool time, mean (SD)											
MVPA (min)	39.2 (1.2)	37.2 (20.8)	46.4 (28.6)	33.5 (17.0)	36.1 (24.5)	43.3 (24.4)	42.0 (24.6)	37.4 (22.0)	33.5 (18.2)	42.3 (27.1)	43.7 (22.9)
LPA (min)	258.8 (45.5)	258.5 (45.4)	259.8 (45.9)	240.3 (42.3)	247.6 (42.0)	266.9 (45.5)	285.4 (37.3)	226.7 (38.7)	248.6 (38.4)	273.2 (41.8)	287.7 (37.7)
Steps (counts)	7343 (116)	7253 (2219)	7674 (2252)	6447 (1734)	6469 (1793)	7425 (1808)	10007 (2589)	6058 (1916)	6630 (1540)	7675 (1725)	9053 (2400)
ST (min)	177.5 (2.4)	181.9 (45.2)	161.1 (44.0)	175.3 (45.7)	177.5 (36.4)	180.9 (53.5)	171.4 (29.4)	164.7 (42.0)	178.3 (42.5)	182.7 (50.7)	184.6 (45.3)
Wear time (min)	446.1 (61.8)	448.6 (63.8)	437.1 (53.3)	419.9 (54.7)	431.2 (44.1)	462.2 (69.7)	469.0 (46.6)	401.5 (55.5)	431.2 (39.6)	467.6 (58.9)	485.8 (54.9)
Preschool time (min)	475.6 (62.1)	477.8 (64.2)	467.4 (53.6)	449.3 (54.5)	461.3 (45.2)	491.2 (70.1)	499.0 (46.6)	429.0 (53.7)	460.5 (40.1)	498.3 (58.8)	516.2 (54.8)
Parental characteristics											
Education, n (%)	N = 337	N = 265	N = 72	N = 87	N = 64	N = 140	N = 46	N = 81	N = 84	N = 88	N = 84
University	271 (80.4)	214 (80.8)	57 (79.2)	73 (83.9)	52 (81.3)	106 (75.7)	40 (87.0)	70 (86)	66 (79)	66 (75)	69 (82)

Abbreviations: BMI = body mass index, MVPA = moderate to vigorous physical activity, LPA = light physical activity, ST = sedentary time, Q1-4 = quartile 1–4.

### Associations between formalized preschool policy and children’s PA, steps and ST

**Fig 2:**
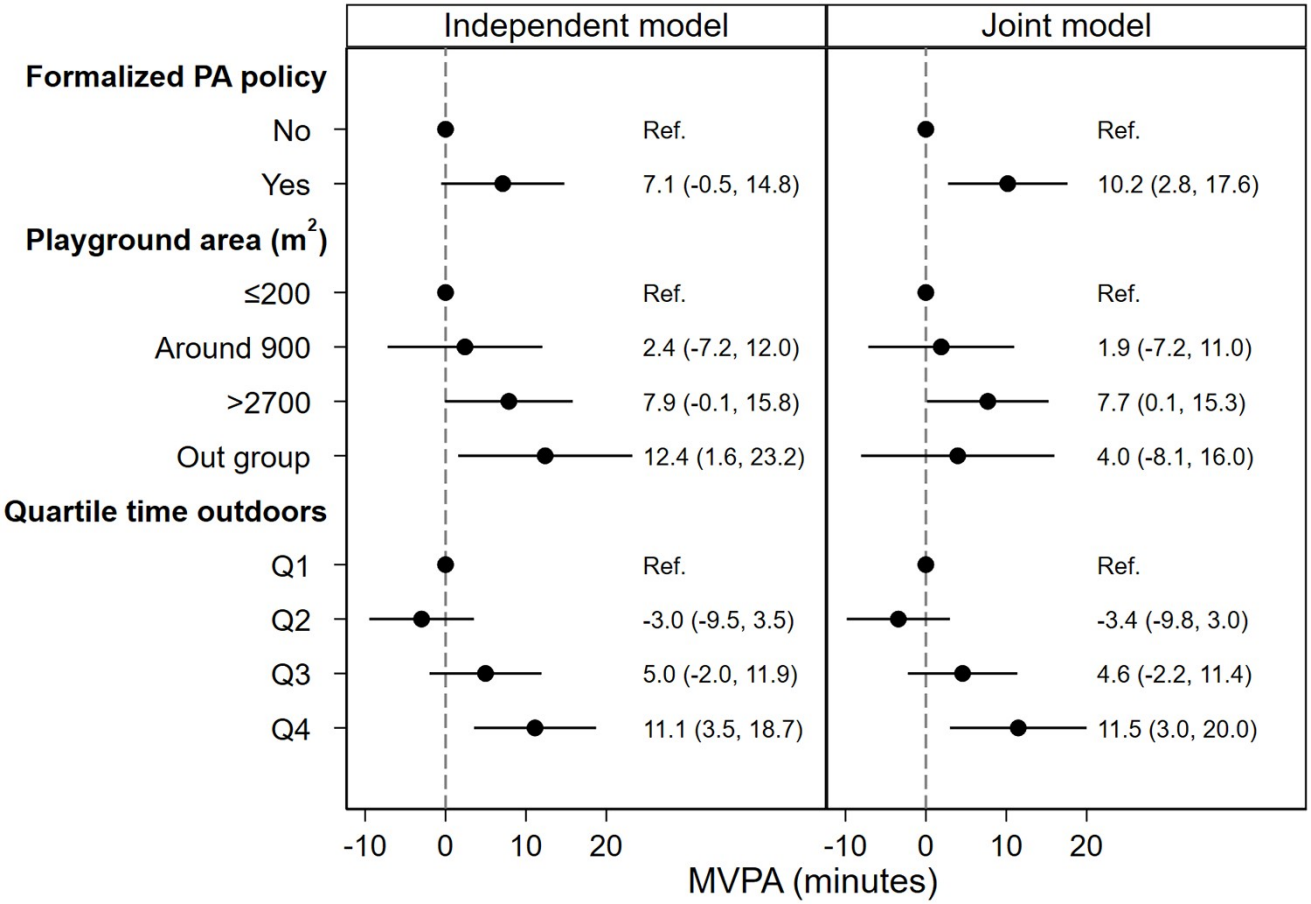
Association between predictors and children’s MVPA during preschool time. Both models are adjusted for age, sex and BMI category. Abbreviations: PA = physical activity, MVPA = moderate to vigorous physical activity, Q1-4 = quartile 1–4, BMI = body mass index.

**Fig 3:**
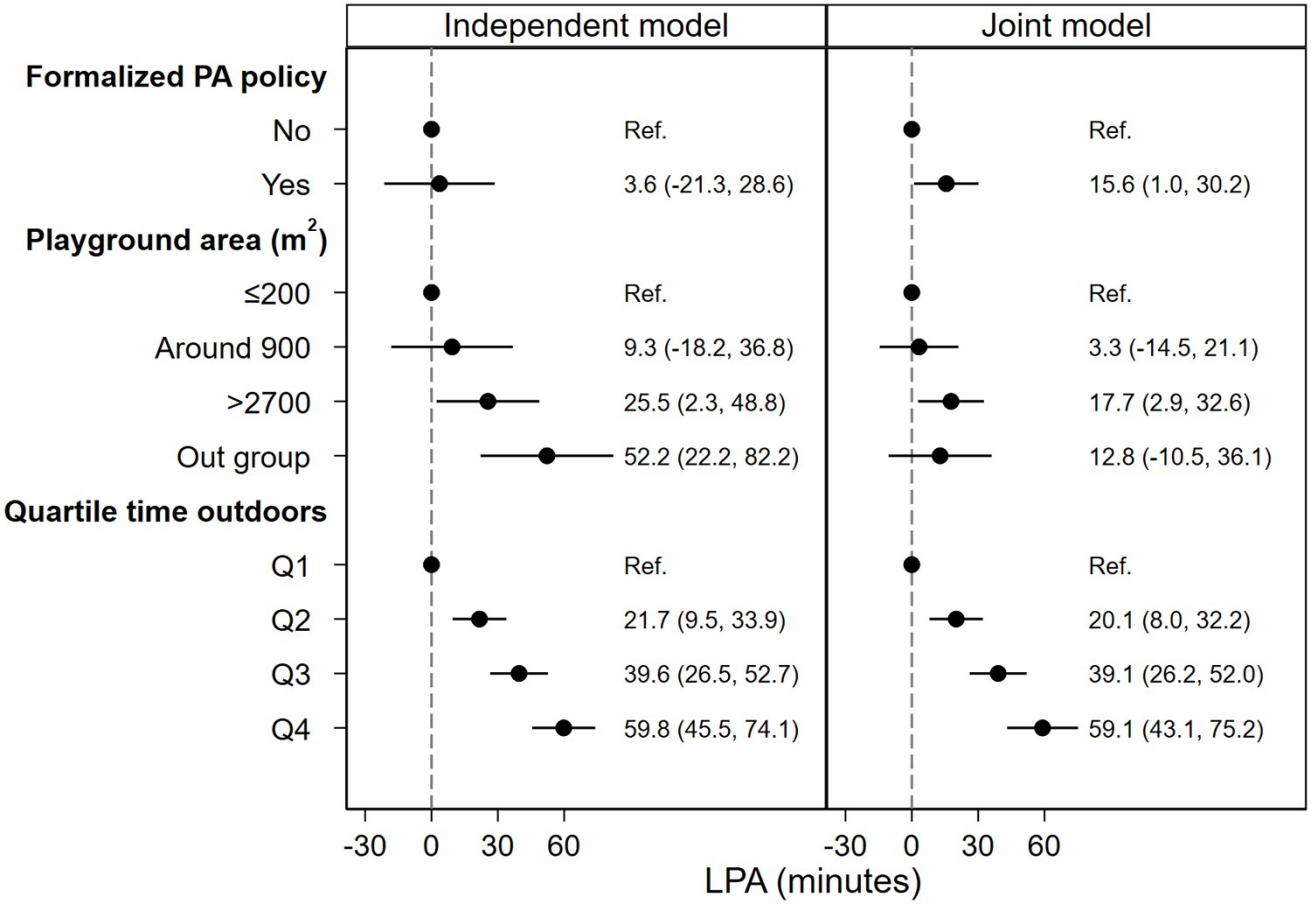
Association between predictors and children’s LPA during preschool time. Both models are adjusted for age, sex and BMI category. Abbreviations: PA = physical activity, LPA = light physical activity, Q1-4 = quartile 1–4, BMI = body mass index.

**Fig 4:**
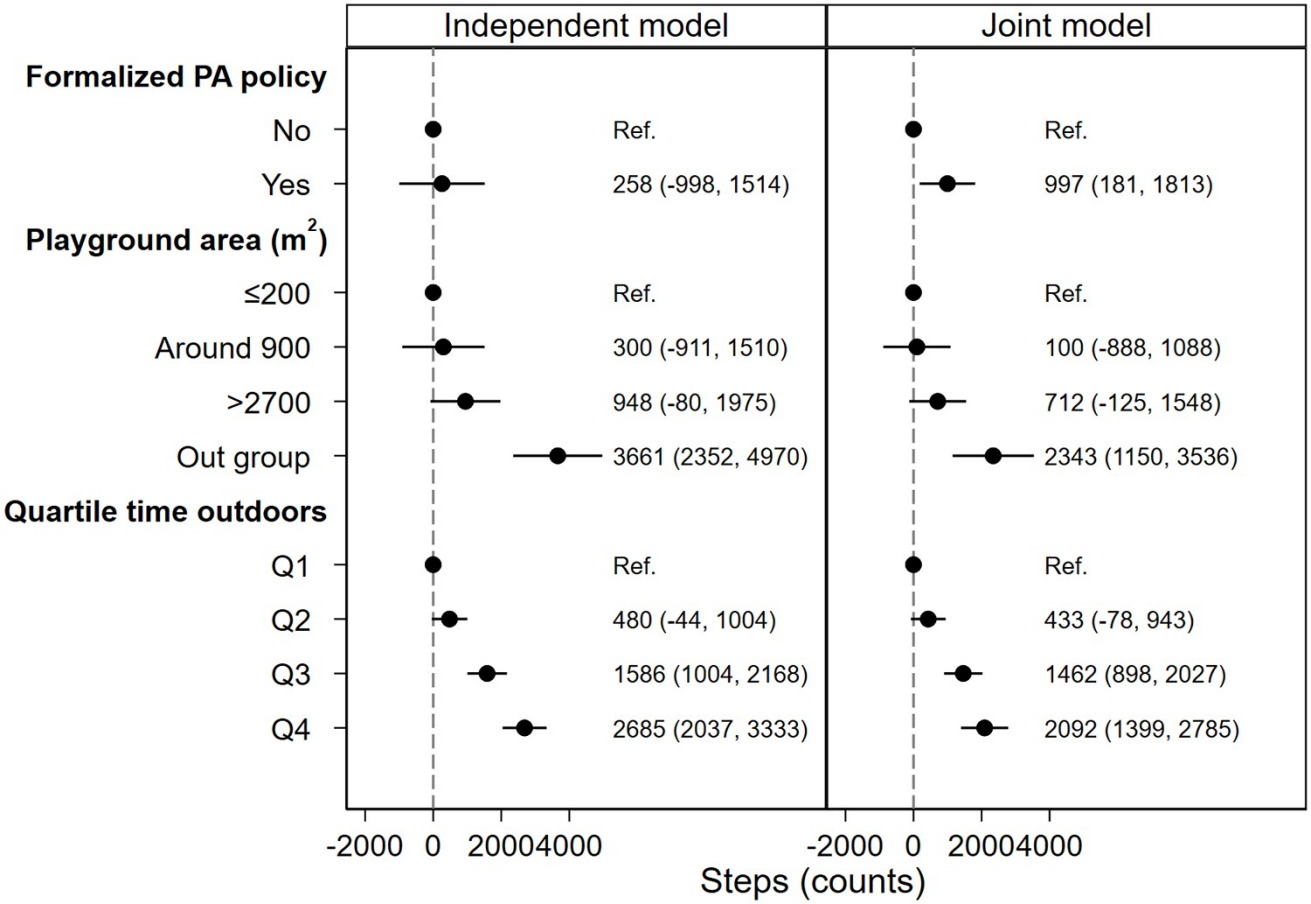
Association between predictors and children’s steps during preschool time. Both models are adjusted for age, sex and BMI category. Abbreviations: Q1-4 = quartile 1–4, BMI = body mass index.

**Fig 5:**
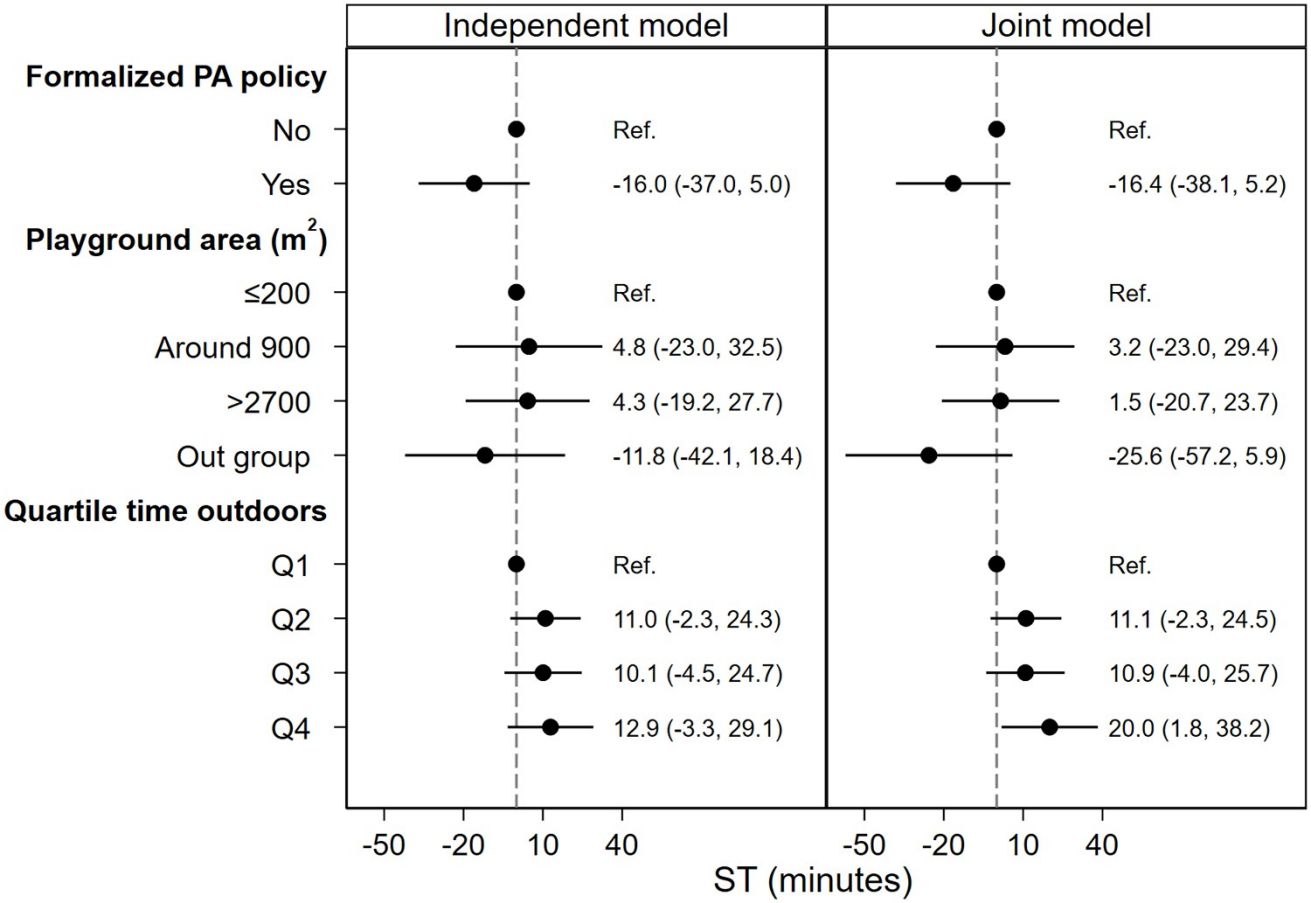
Association between predictors and children’s ST during preschool time. Both models are adjusted for age, sex and BMI category. Abbreviations: ST = sedentary time, Q1-4 = quartile 1–4, BMI = body mass index.

When formalized preschool policy was analyzed as the only predictor in the independent models, the effect of formalized preschool PA policy was hardly manifested (Figs 2–5, independent model). However, when all predictors were analyzed simultaneously (Figs 2–5, joint model), i.e. the direct association between each predictor and outcome, assuming all other predictors constant, children spent 10.2 (95% CI: 2.8, 17.6) minutes more in MVPA (Fig 2, joint model), 15.6 (95% CI: 1, 30.2) minutes more in LPA (Fig 3, joint model) and acquired 997 (95% CI: 181, 1813) more steps (Fig 4, joint model) in preschools with formalized PA policies compared with preschools with no such policies.

### Associations between preschool playground area and children’s PA, steps and ST

As is shown in Figs 2–4, the independent model shows dose-response association between preschool playground area and children’s PA and steps. However, this dose-response association is not displayed when the preschool playground area is analyzed with other predictors simultaneously.

### Associations between time spent outdoors and children’s PA, steps and ST

In the independent model where time spent outdoors was analyzed as the only predictor for the association with children’s PA outcomes, children in the uppermost quartile of time spent outdoors (Q4) spend 11.1 (95% CI: 3.5, 18.7) minutes more in MVPA (Fig 2, independent model), 59.8 (95% CI: 45.5, 74.1) minutes more in LPA (Fig 3, independent model) and acquired 2685 (95% CI: 2037, 3333) more steps compared with children in the lowermost quartile of time spent outdoors (Q1) (Fig 4, independent model). Moreover, when analyzed with all predictors and confounders simultaneously, children in the uppermost quartile of time spent outdoors (Q4) spend 11.5 (95% CI: 3.0, 20.0) minutes more in MVPA (Fig 2, joint model), 59.1 (95% CI: 43.1, 75.2) minutes more in LPA (Fig 3, joint model) and acquired 2092 (95% CI: 1399, 2785) more steps (Fig 4, joint model) compared with children in the lowermost quartile of time spent outdoors (Q1).

### Association between preschool teachers’ PA and children’s PA

**Fig 6:**
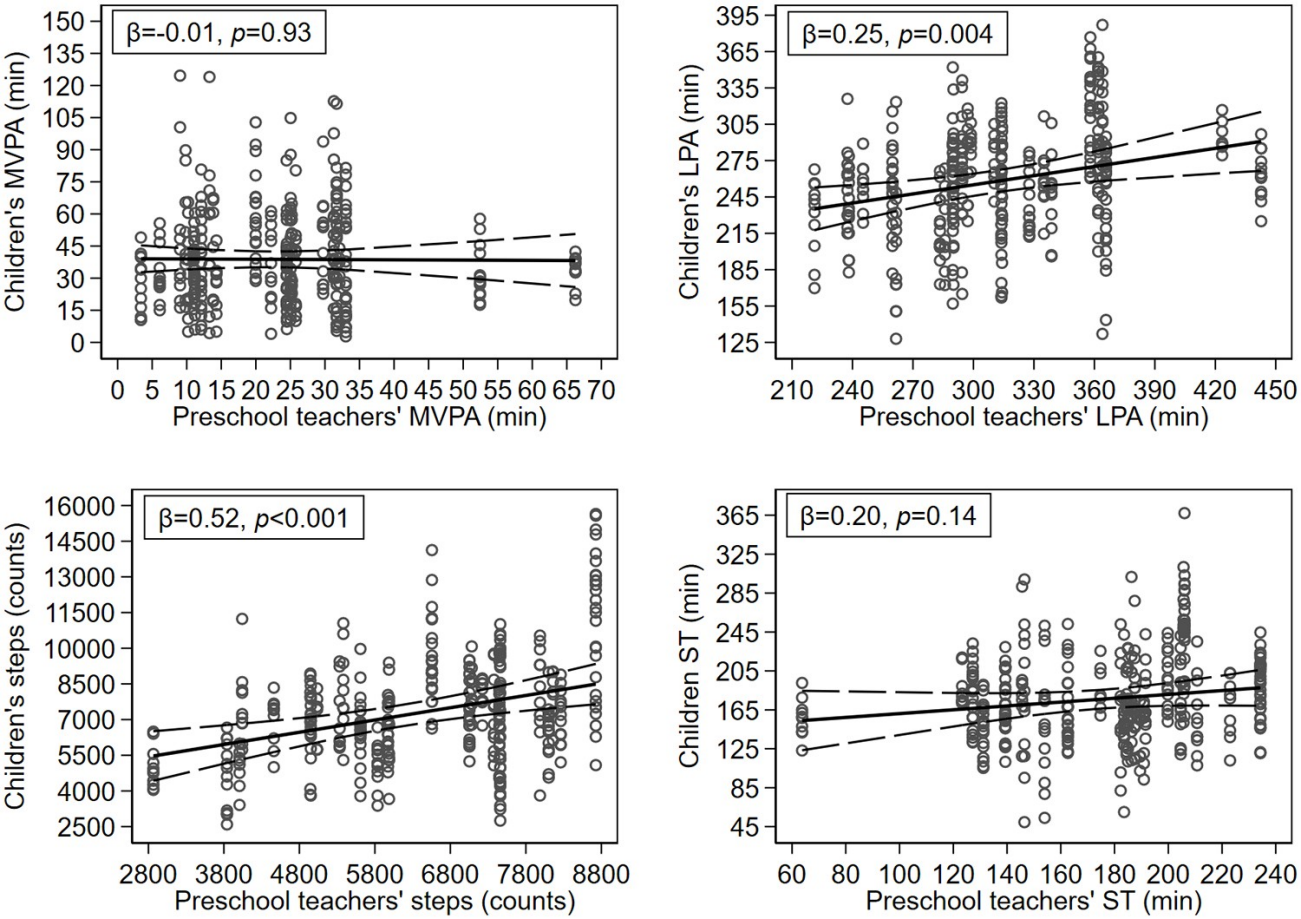
Association between preschool teachers’ and children’s PA and ST during preschool time. Abbreviations: PA = physical activity, MVPA = moderate to vigorous physical activity, LPA = light physical activity, ST = sedentary time.

Fig 6 illustrates the association between preschool teacher’s aggregated levels of MVPA, LPA, steps and ST with children’s levels of MVPA, LPA, steps and ST on an individual level. Both preschool teacher’s aggregated levels of LPA and steps were statistically significant associated with children’s individual levels of LPA (*ß* = 0.25, *P* = 0.004) and steps (*ß* = 0.52, *P*<0.001). However, there was no statistically significant association between preschool teachers aggregated levels of MVPA and ST and children’s levels of MVPA (*ß* = -0.01, *P* = 0.93) and ST (*ß* = 0.20, *P* = 0.14).

### Sensitivity analyses

In sensitivity analyses, we estimated the intra class correlation of each model shown in S2 Table. We observed small to modest ICC for all models (and all outcomes); there was a small ICC for MVPA, and somewhat higher for Steps, LPA and ST (S3 Table). In addition, we ran all models included in S2 Table with additional adjustment made for parental education in a subset population of children (n = 337) (S4 Table). S4 Table shows that none of the estimates are affected, to any substantial extent, by the introduction of parental education as a covariate. Finally, we ran descriptive analyses comparing children in the analytical sample (n = 369) with children with incomplete accelerometer data or missing data on anthropometrics and or missing data on time spent in- and outdoors (n = 35) (S5 Table). As shown in S5 Table, children excluded from analyses, due to missing data, had a higher prevalence of obesity (33% vs. 2%) and lower PA level. The excluded sample were generally distributed across the preschools except for that one preschool contributed to 11 (31.4%) exclusions due to loss of sub-report document for in/out report with information on preschool time and time spent outdoors. This preschool had the smallest preschool playground and did not have a formalized policy.

## Discussion

The current study examined associations between preschool playground size, formalized PA policies, time spent outdoors and preschool teacher’s levels of PA with children’s levels of PA and ST at preschool. Our findings showed that preschool characteristics such as formalized PA policies and more time spent outdoors were positively associated with children’s levels of PA. Moreover, preschool teachers aggregated levels of LPA and steps were statistically significant associated with children’s individual levels of LPA and steps. However, preschool playground size showed no significant association with children’s levels of PA. These findings may be of importance for promoting children’s PA during preschool hours and intervention development.

### Comparison with previous research

Our finding that time spent outdoors was positively associated with children’s levels of PA is supported by previous systematic reviews on positive associations between time outdoors with PA [37] and negative associations with ST [38]. However, most previous studies have relied on potentially biased self-reported levels of PA or retrospective information on time spent in- and outdoors during preschool hours. One small (n = 46) observational study with objectively measured PA and GPS-assessed time spent outdoors showed that children were approximately twice as active and less sedentary when comparing outdoor verses indoor time in a preschool setting [39]. The association in the aforementioned study is somewhat stronger than the 68% more MVPA and 37% less ST accumulated during time spent outdoors compared to indoors we observed in the current study. However, the study by Tandon et al. had a more precise measure of the exposure, i.e. time spend in- and outdoors (GPS vs. preschool teacher reports), which to some extent may explain the observed differences between the two studies.

There is limited evidence that PA policies alone positively stimulate PA and reduce ST in preschool children [40]. In general, PA interventions in preschool settings generate small to moderate effect on children’s MVPA, where multicomponent interventions including structured outdoor activity are most effective [13]. However, a review on the promotion of PA in preschool children [41] highlights the importance of implementing policies concerning PA in preschools to promote children’s levels of PA during preschool hours. Findings in the current study that children spent 10.2 minutes more in MVPA during preschool hours in preschools having formalized PA policies compared with preschools with no such policies, further supports the importance of implementing formalized PA policies.

Systematic review data indicate that preschool playground size and playground software characteristics e.g. play equipment are associated with levels of PA in preschool children [42]. Hence, having enough space to play and having favorable playground conditions may be sufficient for preschool children to be physically active. However, these observational associations have not been reinforced in the few existing interventions studies. In the current study, we found a dose-response association between playground size and children’s levels of PA during preschool hours. However, this association was deteriorated when the association between playground size and PA was evaluated together with PA policy and time outdoors. Thus, the association between playground size and children’s PA may partly be explained by PA policy and time spent outdoors. Nevertheless, more complex relationships may exist. For example, it is possible that these correlating factors interact, and such interactions may be of relevance for PA. However, the potential complex interplay was not explored due to the limited sample size and the cross-sectional design of this study, future studies that perform such detailed investigations are warranted.

Preschool teachers’ individual attitudes and behaviors may play an important role in promoting preschool children’s PA [43]. However, most previous research on the topic are based on qualitative approaches. Thus far, only one Norwegian study has explored accelerometer assessed associations between preschool teachers’ and children’s levels of MVPA during preschool hours [44]. This study demonstrated that there was a statistically significant association between preschool teacher’s aggregated levels of MVPA and preschool children’s individual levels of MVPA. In contrast, we were unable to detect any such association in the current study. However, we observed a statistically significant association between preschool teachers aggregated levels of LPA and steps and children’s individual levels of LPA and steps. The discrepancy between these studies may to some extent be explained by differences in approaches used to classify MVPA intensity in both preschool children and preschool teachers.

### Strengths and limitations

The current study possesses several strengths. First, PA in both preschool children and teachers were assessed objectively with accelerometers during all preschool hours. Thus, limiting certain biases associated with self-reported measures, e.g. social desirability and recall difficulties. Second, the detailed in- and outdoor reports enabled us to match accelerometer data with children’s in- and outdoor time with high resolution (in 30-minute intervals). Third, the study included a large number of participants in preschools with different environmental characteristics (e.g. playground size). Finally, we used a validated instrument (EPAO-SR) to assess preschool characteristics, e.g. PA policies.

Nevertheless, the current study has several limitations that need consideration. First, both preschool characteristics, assessed via the EPAO-SR, and the in- and outdoor reporting relied on preschool teacher’s self-reporting, which have several inherited biases. In addition, formalized PA policy was dichotomized into yes/no which may have disregarded the influence of policy’s specific content on PA outcomes [19, 21]. Investigation further into content of formalized policy was hindered by the limited number of preschools that had formalized policy and unavailability of implementation information. Second, the geographical distribution of preschools was limited to a small area in Stockholm with a homogenous socioeconomic distribution. Furthermore, our sensitivity analysis showed that children with incomplete data had a higher prevalence of obesity and lower PA level compared with those included in the main analyses. However, it is important to note that few observations (25 observations for obesity status and 8 observations for PA level) in the excluded sample due to missing value may limit the representativeness of this result in the excluded sample. Further, one preschool, with no formalized policy and a playground ≤ 200m^2^, contributed to 31.4% of the excluded participants due to loss of sub-document of in/out report with information on preschool time and time spent outdoors. This is of importance in relation to the center-level influence on missing data. Nevertheless, the constricted socioeconomic and body size distribution limit the generalizability of findings in the current study. Third, although accelerometry is considered as a preferable measurement of PA among preschool children in free-living conditions, it is unable to detect all PA when attached to the hip, e.g. cycling or upper-body movements [45]. Thus, some of preschoolers PA during preschool hours may not be captured, which may impact or estimates of PA and ST. Forth, information regarding child and preschool teacher associations in PA may have been diminished due to aggregating preschool teacher levels of PA within the preschools. Moreover, with the cross-sectional nature of data, it is not possible to conclude if preschool teachers PA affect children’s PA or vice versa. Finally, by using the normal filter to process accelerometer data we may have underestimated the number of steps taken during preschool hours [46]. In addition, a 60s epoch was adopted in accelerometer data analysis while a shorter epoch length has been suggested to suit the young children’s sporadic moving nature [31]. However, studies also suggest that scaling the cut-offs to suit a different epoch may be problematic [47]. The accuracy of cut-offs to classify PA level may be optimal when they are applied under the same epoch setting as the calibration setting that developed these cut-offs [47, 48]. Therefore, accelerometer data was analyzed in 60 s epoch strictly following the epoch length used in the validation study [32].

## Conclusions

The current study showed that modifiable preschool characteristics such as formalized PA policies and more time spent outdoors were positively associated with children’s objectively measured levels of PA during preschool hours. For promoting children’s PA during preschool hours, preschools should consider incorporating formalized PA policies and aim to increase the daily amount of time spent outdoors. However, given the cross-sectional nature of the current study, these findings need further examination, preferably using experimental research designs.

## Supporting information

S1 Table Cross tabulation of teachers' PA and children' s PA. High and low are classified by the median of the respective teacher PA variable Abbreviations: PA = physical activity, MVPA = moderate to vigorous physical activity, LPA = light physical activity, ST = sedentary time.
(DOCX)

S2 Table Associations between predictors and physical activity indicators during preschool time (n = 369). Model 1 = crude model each predictor independently, Model 2 = Model 1 adjusted for age, sex, BMI category Model 3 = all predictors jointly, Model4 = Model 3 adjusted for age, sex, BMI category Abbreviations: PA = physical activity, BMI = body mass index, MVPA = moderate to vigorous physical activity, LPA = light physical activity, ST = sedentary time, Q1-4 = quartile 1–4 Reference level: Formalized PA policy = No, Playground area = ≤200 m^2^, Time spend outdoors = Q1.
(DOCX)

S3 Table Preschool-level cluster effect (intra class correlation) in each linear mixed model. Model 1 = crude model each predictor independently, Model 2 = Model 1 adjusted for age, sex, BMI category Model 3 = all predictors jointly, Model 4 = Model 3 adjusted for age, sex, age, BMI category Abbreviations: MVPA = moderate to vigorous physical activity, LPA = light physical activity, ST = sedentary time Reference level: Formalized PA policy = No, Playground area = ≤200 m^2^, Time spend outdoors = Q1.
(DOCX)

S4 Table Associations between predictors and physical activity indicators during preschool time (n = 337). Model 1 = crude model each predictor independently, Model 2 = Model 1 adjusted for age, sex, BMI category and parental education Model 3 = all predictors jointly, Model4 = Model 3 adjusted for age, sex, age, BMI category and parental education Abbreviations: PA = physical activity, BMI = body mass index, MVPA = moderate to vigorous physical activity, LPA = light physical activity, ST = sedentary time, Q1-4 = quartile 1–4 Reference level: Formalized PA policy = No, Playground area = ≤200 m2, Time spend outdoors = Q1.
(DOCX)

S5 Table Comparison of descriptive characteristics between analytical dataset and excluded observations. PA = physical activity, BMI = body mass index, MVPA = moderate to vigorous physical activity, LPA = light physical activity, ST = sedentary time, Q1-4 = quartile 1–4.
(DOCX)

S6 Table Policy content in seven preschool reported formalized policy. (DOCX)
