# Supplementary material for: Preschool environment and preschool teacher’s physical activity and their association with children’s activity levels at preschool
Source: PLoS One. 2020 Oct 15;15(10):e0239838. doi: 10.1371/journal.pone.0239838 (PMC7561096; doi:10.1371/journal.pone.0239838)
Supplement: S1 Table — High and low are classified by the median of the respective teacher PA variable Abbreviations: PA = physical activity, MVPA = moderate to vigorous physical activity, LPA = light physical activity, ST = sedentary time. (DOCX) [file pone.0239838.s001.docx]

**S1 Table. Cross tabulation of teachers' PA and children' s PA.**

|  | Teacher’s PA aggregated at preschool level | | | | | | | |
| --- | --- | --- | --- | --- | --- | --- | --- | --- |
|  | MVPA | | LPA | | Steps | | ST | |
|  | high | low | high | low | high | low | high | Low |
| Children PA, mean (SD) | N=192 | N=177 | N=185 | N=184 | N=185 | N=184 | N=182 | N=187 |
| MVPA (min) | 38.5 (22.4) | 39.8 (23.6) |  |  |  |  |  |  |
| LPA (min) |  |  | 269.1 (47.3) | 248.4 (41.3) |  |  |  |  |
| Steps (counts) |  |  |  |  | 7918 (2371) | 6765 (1916) |  |  |
| ST (min) |  |  |  |  |  |  | 188.0 (48.1) | 167.2 (40.8) |

High and low are classified by the median of the respective teacher PA variable

Abbreviations: PA = physical activity, MVPA = moderate to vigorous physical activity, LPA = light physical activity, ST = sedentary time
