# Supplementary material for: Preschool environment and preschool teacher’s physical activity and their association with children’s activity levels at preschool
Source: PLoS One. 2020 Oct 15;15(10):e0239838. doi: 10.1371/journal.pone.0239838 (PMC7561096; doi:10.1371/journal.pone.0239838)
Supplement: S2 Table — Model 1 = crude model each predictor independently, Model 2 = Model 1 adjusted for age, sex, BMI category Model 3 = all predictors jointly, Model4 = Model 3 adjusted for age, sex, BMI category Abbreviations: PA = physical activity, BMI = body mass index, MVPA = moderate to vigorous physical activity, LPA = light physical activity, ST = sedentary time, Q1-4 = quartile 1–4 Reference level: Formalized PA policy = No, Playground area = ≤200 m2, Time spend outdoors = Q1. (DOCX) [file pone.0239838.s002.docx]

**S2 Table. Associations between predictors and physical activity indicators during preschool time (n=369).**

|  | Model 1 | | Model 2 | | Model 3 | | Model 4 | |
| --- | --- | --- | --- | --- | --- | --- | --- | --- |
|  | Coef. | CI 95% | Coef. | CI 95% | Coef. | CI 95% | Coef. | CI 95% |
| MVPA (min) | | | | | | | | |
| Formalized PA Policy | | | | | | | | |
| Yes | 8.5 | 0.6, 16.3 | 7.1 | -0.6, 14.8 | 12.0 | 3.4, 20.7 | 10.2 | 2.8, 17.6 |
| Playground area (m^2^) | | | | | | | | |
| Around 900 | 0.4 | -10.6, 11.4 | 2.4 | -7.2, 12.0 | 0.2 | -10.4, 10.8 | 1.9 | -7.2, 11.0 |
| > 2700 | 8.3 | -0.8, 17.3 | 7.9 | -0.1, 15.8 | 8.4 | -0.4, 17.3 | 7.7 | 0.1, 15.3 |
| Out group | 13.5 | 1.2, 25.9 | 12.4 | 1.6, 23.2 | 4.6 | -9.2, 18.3 | 4.0 | -8.1, 16.0 |
| Time spent outdoors | | | | | | | | |
| Q2 | -5.4 | -12.5, 1.7 | -3.0 | -9.5, 3.5 | -5.9 | -12.9, 1.1 | -3.4 | -9.8, 3.0 |
| Q3 | 5.2 | -2.5, 12.1 | 5.0 | -2.0, 11.9 | 4.6 | -2.9, 12.1 | 4.6 | -2.2, 11.4 |
| Q4 | 11.9 | 3.5, 20.3 | 11.1 | 3.5 ,18.7 | 11.6 | 2.2, 20.9 | 11.5 | 3.0, 20.0 |
| LPA (min) | | | | | | | | |
| Formalized PA Policy | | | | | | | | |
| Yes | 4.4 | -19.9, 28.6 | 3.6 | -21.3, 28.6 | 16.0 | 1.6, 30.4 | 15.6 | 1.0, 30.2 |
| Playground area (m^2^) | | | | | | | | |
| Around 900 | 8.1 | -19.3, 35.4 | 9.3 | -18.2, 36.8 | 2.8 | -14.8, 20.4 | 3.3 | -14.5, 21.1 |
| > 2700 | 26.2 | 3.1, 49.3 | 25.5 | 2.3, 48.8 | 18.7 | 4.0, 33.4 | 17.8 | 2.9, 32.6 |
| Out group | 53.6 | 23.8, 83.4 | 52.2 | 22.2, 82.2 | 12.6 | -10.5, 35.7 | 12.8 | -10.5, 36.1 |
| Time spent outdoors | | | | | | | | |
| Q2 | 20.3 | 8.2, 32.5 | 21.7 | 9.5, 33.9 | 18.7 | 6.7, 30.8 | 20.1 | 8.0, 32.2 |
| Q3 | 38.80 | 25.7, 51.9 | 39.6 | 26.5, 52.7 | 38.3 | 25.4, 51.1 | 39.1 | 26.2, 52.0 |
| Q4 | 59.36 | 45.1, 73.7 | 59.8 | 45.5, 74.1 | 58.8 | 42.8, 74.8 | 59.1 | 43.1, 75.2 |
| Steps (counts) | | | | | | | | |
| Formalized PA Policy | | | | | | | | |
| Yes | 387 | -833, 1606 | 258 | -998, 1514 | 1162 | 264, 2060 | 997 | 181, 1813 |
| Playground area (m^2^) | | | | | | | | |
| Around 900 | 89 | -1210, 1388 | 300 | -911, 151 | -68 | -1156, 1020 | 100 | -888, 1088 |
| > 2700 | 988 | -118, 2094 | 948 | -80, 1975 | 780 | -144, 1704 | 712 | -125, 1548 |
| Out group | 3950 | 2549, 5351 | 3661 | 2352, 497 | 2564 | 1266, 3862 | 2343 | 1150, 3536 |
| Time spent outdoors | | | | | | | | |
| Q2 | 348 | -207, 903 | 480 | -44, 1004 | 285 | -253, 824 | 433 | -78, 943 |
| Q3 | 1614 | 999, 2229 | 1586 | 1004, 2168 | 1454 | 856, 2052 | 1462 | 898, 2027 |
| Q4 | 2783 | 2097, 3469 | 2685 | 2037, 3333 | 2072 | 1337, 2806 | 2092 | 1399, 2785 |
| ST (min) | | | | | | | | |
| Formalized PA Policy | | | | | | | | |
| Yes | -16.7 | -38.3, 4.8 | -16.0 | -37.0, 5.0 | -17.8 | -40.5, 4.9 | -16.5 | -38.1, 5.2 |
| Playground area (m^2^) | | | | | | | | |
| Around 900 | 7.7 | -21.3, 36.8 | 4.8 | -23.0, 32.5 | 5.7 | -21.8, 33.1 | 3.2 | -23.0, 29.4 |
| > 2700 | 3.3 | -21.4, 27.9 | 4.3 | -19.2, 27.7 | 0.1 | -23.3, 23.4 | 1.5 | -20.7, 23.7 |
| Out group | -14.8 | -46.4, 16.8 | -11.8 | -42.1, 18.4 | -26.9 | -59.7, 5.6 | -25.6 | -57.2, 5.9 |
| Time spent outdoors | | | | | | | | |
| Q2 | 13.9 | 0.3, 27.4 | 11.0 | -2.3, 24.3 | 14.0 | 0.3, 27.6 | 11.1 | -2.3, 24.5 |
| Q3 | 10.4 | -4.5, 25.3 | 10.1 | -4.5, 24.7 | 11.3 | -3.8, 26.5 | 10.9 | -4.0, 25.7 |
| Q4 | 11.8 | -4.8, 28.3 | 12.9 | -3.3, 29.1 | 19.5 | 0.9, 38.1 | 20.0 | 1.9, 38.2 |

Model 1 = crude model each predictor independently, Model 2 = Model 1 adjusted for age, sex, BMI category

Model 3= all predictors jointly, Model4 = Model 3 adjusted for age, sex, BMI category

Abbreviations: PA = physical activity, BMI = body mass index, MVPA = moderate to vigorous physical activity,

LPA = light physical activity, ST = sedentary time, Q1-4 = quartile 1-4

Reference level: Formalized PA policy = No, Playground area = ≤200 m^2^, Time spend outdoors = Q1
