# Supplementary material for: Preschool environment and preschool teacher’s physical activity and their association with children’s activity levels at preschool
Source: PLoS One. 2020 Oct 15;15(10):e0239838. doi: 10.1371/journal.pone.0239838 (PMC7561096; doi:10.1371/journal.pone.0239838)
Supplement: S3 Table — Model 1 = crude model each predictor independently, Model 2 = Model 1 adjusted for age, sex, BMI category Model 3 = all predictors jointly, Model 4 = Model 3 adjusted for age, sex, age, BMI category Abbreviations: MVPA = moderate to vigorous physical activity, LPA = light physical activity, ST = sedentary time Reference level: Formalized PA policy = No, Playground area = ≤200 m2, Time spend outdoors = Q1. (DOCX) [file pone.0239838.s003.docx]

**S3 Table Preschool-level cluster effect (Intra class correlation) in each Linear Mixed Model.**

|  | Model 1 |  | Model 2 |  |
| --- | --- | --- | --- | --- |
|  | ICC | CI 95% | ICC | CI 95% |
| MVPA | | | | |
| Policy | 0.078 | 0.028, 0.198 | 0.096 | 0.038, 0.220 |
| Time outdoor | 0.152 | 0.070, 0.298 | 0.130 | 0.056, 0.274 |
| Playground size | 0.128 | 0.048, 0.299 | 0.108 | 0.040, 0.264 |
| LPA | | | | |
| Policy | 0.308 | 0.183, 0.469 | 0.326 | 0.197, 0.488 |
| Time outdoor | 0.137 | 0.062, 0.276 | 0.139 | 0.063, 0.280 |
| Playground size | 0.286 | 0.160, 0.458 | 0.290 | 0.163, 0.461 |
| Steps | | | | |
| Policy | 0.369 | 0.235, 0.524 | 0.424 | 0.282, 0.579 |
| Time outdoor | 0.293 | 0.177, 0.445 | 0.306 | 0.186, 0.460 |
| Playground size | 0.348 | 0.208, 0.520 | 0.337 | 0.202, 0.505 |
| ST | | | | |
| Policy | 0.240 | 0.135, 0.390 | 0.238 | 0.132, 0.391 |
| Time outdoor | 0.245 | 0.140, 0.394 | 0.239 | 0.134, 0.390 |
| Playground size | 0.293 | 0.169, 0.460 | 0.279 | 0.157, 0.446 |
|  | Model 3 |  | Model 4 |  |
|  | ICC | CI 95% | ICC | CI 95% |
| MVPA | 0.112 | 0.040, 0.278 | 0.086 | 0.027, 0.024 |
| LPA | 0.099 | 0.036, 0.243 | 0.103 | 0.038, 0.250 |
| Steps | 0.264 | 0.146, 0.430 | 0.241 | 0.130, 0.423 |
| ST | 0.262 | 0.145, 0.427 | 0.247 | 0.133, 0.413 |

Model 1 = crude model each predictor independently, Model 2 = Model 1 adjusted for age, sex, BMI category Model 3= all predictors jointly, Model4 = Model 3 adjusted for age, sex, age, BMI category

Abbreviations: MVPA = moderate to vigorous physical activity, LPA = light physical activity, ST = sedentary time
