# Supplementary material for: Preschool environment and preschool teacher’s physical activity and their association with children’s activity levels at preschool
Source: PLoS One. 2020 Oct 15;15(10):e0239838. doi: 10.1371/journal.pone.0239838 (PMC7561096; doi:10.1371/journal.pone.0239838)
Supplement: S4 Table — Model 1 = crude model each predictor independently, Model 2 = Model 1 adjusted for age, sex, BMI category and parental education Model 3 = all predictors jointly, Model4 = Model 3 adjusted for age, sex, age, BMI category and parental education Abbreviations: PA = physical activity, BMI = body mass index, MVPA = moderate to vigorous physical activity, LPA = light physical activity, ST = sedentary time, Q1-4 = quartile 1–4 Reference level: Formalized PA policy = No, Playground area = ≤200 m2, Time spend outdoors = Q1. (DOCX) [file pone.0239838.s004.docx]

**S4 Table. Associations between predictors and physical activity indicators during preschool time (n=337).**

|  | Model 1 | | Model 2 | | Model 3 | | Model 4 | |
| --- | --- | --- | --- | --- | --- | --- | --- | --- |
|  | Coef. | CI 95% | Coef. | CI 95% | Coef. | CI 95% | Coef. | CI 95% |
| MVPA (min) | | | | | | | | |
| Formalized PA Policy | | | | | | | | |
| Yes | 8.5 | 1.0, 16.1 | 7.1 | -0.5, 14.6 | 12.0 | 3.6, 20.3 | 10.0 | 2.7, 17.4 |
| Playground area (m^2^) | | | | | | | | |
| Around 900 | 0.5 | -10.2, 11.1 | 3.0 | -6.4, 12.4 | 0.4 | -9.8, 10.5 | 2.7 | -6.1, 11.5 |
| > 2700 | 7.2 | -1.6, 16.1 | 7.4 | -0.4, 15.2 | 7.5 | -1.0, 16.0 | 7.4 | 0.1, 14.8 |
| Out group | 12.0 | -0.1, 24.0 | 11.8 | 1.2, 22.4 | 1.4 | -11.9, 14.7 | 1.7 | -10.0, 13.4 |
| Time spent outdoors | | | | | | | | |
| Q2 | -7.1 | -14.4, 0.2 | -4.8 | -11.5, 1.9 | -7.2 | -14.4, 0.0 | -4.8 | -11.3, 1.8 |
| Q3 | 2.8 | -4.9, 10.5 | 2.5 | -4.5, 9.5 | 2.6 | -4.9, 10.1 | 2.7 | -4.1, 9.5 |
| Q4 | 11.6 | 3.2, 20.0 | 10.8 | 3.2, 18.4 | 12.6 | 3.2, 21.9 | 12.6 | 4.1, 21.1 |
| LPA (min) | | | | | | | | |
| Formalized PA Policy | | | | | | | | |
| Yes | 8.0 | -17.2, 33.1 | 6.9 | -19.9, 33.7 | 20.3 | 5.1, 35.5 | 19.5 | 3.6, 35.4 |
| Playground area (m^2^) | | | | | | | | |
| Around 900 | 6.9 | -21.6, 35.5 | 9.1 | -20.4, 38.5 | 1.7 | -16.8, 20.3 | 2.9 | -16.5, 22.2 |
| > 2700 | 27.0 | 2.9, 51.2 | 27.6 | 2.6, 52.6 | 19.8 | 4.2, 35.4 | 31.9 | 3.7, 36.2 |
| Out group | 55.2 | 24.3, 86.1 | 52.7 | 20.9, 84.6 | 14.0 | -10.0, 37.9 | 25.1 | -11.1, 38.2 |
| Time spent outdoors | | | | | | | | |
| Q2 | 19.7 | 6.9, 32.4 | 20.9 | 8.1, 33.6 | 18.7 | 6.2, 31.2 | 20.0 | 7.4, 32.6 |
| Q3 | 37.0 | 23.4, 50.6 | 37.2 | 23.5, 50.8 | 37.1 | 23.9, 50.3 | 37.5 | 24.2, 50.9 |
| Q4 | 60.5 | 45.6, 75.4 | 60.3 | 45.3, 75.3 | 60.0 | 43.7, 76.4 | 60.3 | 43.8, 76.8 |
| Steps (counts) | | | | | | | | |
| Formalized PA Policy | | | | | | | | |
| Yes | 439 | -797, 1676 | 275 | -1021, 1571 | 1207 | 294, 2120 | 1002 | 171, 1834 |
| Playground area (m^2^) | | | | | | | | |
| Around 900 | 30 | -1282, 1343 | 320 | -910, 1551 | -99 | -1203, 1005 | 151 | -1321, 1862 |
| > 2700 | 905 | -215, 2025 | 910 | -138, 1959 | 727 | -213, 1667 | 715 | -138, 1569 |
| Out group | 3916 | 2500, 5333 | 3656 | 2328, 4984 | 2551 | 1238, 3865 | 2358 | 1151, 3564 |
| Time spent outdoors | | | | | | | | |
| Q2 | 170 | -414, 754 | 253 | -295, 801 | 121 | -443, 685 | 234 | -298, 765 |
| Q3 | 1414 | 777, 2051 | 1330 | 731, 1929 | 1255 | 638, 1872 | 1229 | 649, 1808 |
| Q4 | 2679 | 1972, 3385 | 2547 | 1881, 3212 | 1965 | 1213, 2717 | 1979 | 1271, 2687 |
| ST (min) | | | | | | | | |
| Formalized PA Policy | | | | | | | | |
| Yes | -18.9 | -39.5, 1.7 | -18.9 | -38.2, 0.4 | -20.6 | -42.4, 1.2 | -19.7 | -39.9, 0.4 |
| Playground area (m^2^) | | | | | | | | |
| Around 900 | 9.1 | -18.7, 37.0 | 6.1 | -20.0, 32.1 | 8.2 | -18.2, 34.5 | 5.7 | -18.8, 24.5 |
| > 2700 | 5.8 | -17.8, 29.4 | 5.5 | -16.5, 27.5 | 3.8 | -18.6, 26.2 | 3.8 | -16.8, 12.6 |
| Out group | -10.9 | -41.3, 19.5 | -7.5 | -36.1, 21.0 | -17.6 | -49.3, 14.2 | -17.2 | -47.0, 12.6 |
| Time spent outdoors | | | | | | | | |
| Q2 | 11.6 | -2.4, 25.6 | 8.6 | -5.0, 22.2 | 11.2 | -2.9, 25.3 | 8.1 | -5.6, 21.8 |
| Q3 | 7.4 | -7.8, 22.5 | 7.5 | -7.1, 22.2 | 7.5 | -7.9, 22.9 | 7.4 | -7.4, 22.3 |
| Q4 | 6.2 | -10.5, 23.0 | 8.7 | -7.5, 24.9 | 11.3 | -7.5, 30.1 | 13.4 | -4.8, 31.6 |

Model 1 = crude model each predictor independently, Model 2 = Model 1 adjusted for age, sex, BMI category

and parental education Model 3= all predictors jointly, Model4 = Model 3 adjusted for age, sex, age, BMI category and parental education

Abbreviations: PA = physical activity, BMI = body mass index, MVPA = moderate to vigorous physical activity,

LPA = light physical activity, ST = sedentary time, Q1-4 = quartile 1-4

Reference level: Formalized PA policy = No, Playground area = ≤200 m^2^, Time spend outdoors = Q1
