# Supplementary material for: Preschool environment and preschool teacher’s physical activity and their association with children’s activity levels at preschool
Source: PLoS One. 2020 Oct 15;15(10):e0239838. doi: 10.1371/journal.pone.0239838 (PMC7561096; doi:10.1371/journal.pone.0239838)
Supplement: S5 Table — PA = physical activity, BMI = body mass index, MVPA = moderate to vigorous physical activity, LPA = light physical activity, ST = sedentary time, Q1-4 = quartile 1–4. (DOCX) [file pone.0239838.s005.docx]

**S5 Table. Comparison of descriptive characteristics between analytical dataset and excluded observations.**

|  | Analytical dataset  (N=369) | Excluded  (Total N= 35) | P value |
| --- | --- | --- | --- |
| Individual characteristics |  |  |  |
| Boys, n (%) | (n=369) | (n=35) |  |
|  | 204 (55.3%) | 18 (48%) | 0.66 |
| Age, mean (SD) | (n=369) | (n=35) |  |
|  | 4.7 (0.1) | 4.6 (0.1) | 0.90 |
| …..Body measures | (n=369) | (n=25*) |  |
| BMI, mean (SD) | 15.7 (0.1) | 14.0 (1.4) | 0.01 |
| Overweight, n (%) | 25 (6.8%) | 2 (7.4%) | <0.001 |
| Obesity, n (%) | 8 (2.2%) | 9 (33.3%) |  |
| School characteristics |  |  |  |
| Has formalized PA policy, n (%) | (n=369) | (n=35) |  |
|  | 79 (27.2%) | 3 (8.6%) | 0.07 |
| Playground area (m^2^), n (%) | (n=369) | (n=35) |  |
| ≤200 | 98 (26.5%) | 17 (48.6%) | <0.001 |
| Around 900 | 69 (18.7%) | 12 (34.3%) |  |
| >2700 | 151 (40.9%) | 5 (14.3%) |  |
| Out group | 51 (13.8%) | 1 (2.9%) |  |
| Time spent outdoors (min), mean (SD) | (n=369) | (n=8**) |  |
|  | 206.4 (5.6) | 127.5 (36.2) | 0.04 |
| Child PA during preschool time, mean (SD) | (n=369) | (n=8**) |  |
| MVPA (min) | 39.2 (1.2) | 38.7 (9.6) | 0.95 |
| LPA (min) | 258.8 (45.5) | 220.3 (51.5) | 0.02 |
| Steps (counts) | 7343 (116) | 5775 (786) | 0.05 |
| ST (min) | 177.5 (2.4) | 174.8 (18.7) | 0.87 |

PA = physical activity, BMI = body mass index, MVPA = moderate to vigorous physical activity, LPA = light physical activity, ST = sedentary time, Q1-4 = quartile 1-4

*Only 25 participants in the excluded datasets had available BMI data

**Only 8 participants in the excluded datasets had available preschool time and time spent outdoors data
