# Supplementary material for: Preschool environment and preschool teacher’s physical activity and their association with children’s activity levels at preschool
Source: PLoS One. 2020 Oct 15;15(10):e0239838. doi: 10.1371/journal.pone.0239838 (PMC7561096; doi:10.1371/journal.pone.0239838)
Supplement: S6 Table — (DOCX) [file pone.0239838.s006.docx]

**S6. Table Policy content in seven preschool reported formalized policy.**

| Formalized policy content | Number of preschools reported such policy |
| --- | --- |
| Minimal daily time spent outdoors | 7 |
| Regular organized physical activity | 5 |
| Limited longer sedentary period | 2 |
| Teachers promote physical activity while being outdoors | 6 |
| Parents drop off/pick up children at preschool outdoors | 7 |
| Teachers participate in children’s active play | 6 |
| Teachers avoid using mobile phone/chatting with colleague while being outdoors | 7 |
