# Supplementary material for: Preschool environment and preschool teacher’s physical activity and their association with children’s activity levels at preschool
Source: PLoS One. 2020 Oct 15;15(10):e0239838. doi: 10.1371/journal.pone.0239838 (PMC7561096; doi:10.1371/journal.pone.0239838)
Supplement: Peer review history

# Peer review: Decision Letter 0

**DOI:** 10.1371/journal.pone.0239838.r001

**Authors:** David Paul

22 May 2020

PONE-D-20-01800

Preschool environment and preschool teacher’s physical activity and their association with children’s activity levels at preschool

PLOS ONE

Dear Dr. Chen,

Thank you for submitting your manuscript to PLOS ONE. After careful consideration, we feel that it has merit but does not fully meet PLOS ONE’s publication criteria as it currently stands. Therefore, we invite you to submit a revised version of the manuscript that addresses the points raised during the review process.

Please submit your revised manuscript by Jul 05 2020 11:59PM. If you will need more time than this to complete your revisions, please reply to this message or contact the journal office at plosone@plos.org. When you're ready to submit your revision, log on to https://www.editorialmanager.com/pone/ and select the 'Submissions Needing Revision' folder to locate your manuscript file.

Please include the following items when submitting your revised manuscript:

- A rebuttal letter that responds to each point raised by the academic editor and reviewer(s). You should upload this letter as a separate file labeled 'Response to Reviewers'.
- A marked-up copy of your manuscript that highlights changes made to the original version. You should upload this as a separate file labeled 'Revised Manuscript with Track Changes'.
- An unmarked version of your revised paper without tracked changes. You should upload this as a separate file labeled 'Manuscript'.

If you would like to make changes to your financial disclosure, please include your updated statement in your cover letter. Guidelines for resubmitting your figure files are available below the reviewer comments at the end of this letter.

If applicable, we recommend that you deposit your laboratory protocols in protocols.io to enhance the reproducibility of your results. Protocols.io assigns your protocol its own identifier (DOI) so that it can be cited independently in the future. For instructions see: http://journals.plos.org/plosone/s/submission-guidelines#loc-laboratory-protocols

We look forward to receiving your revised manuscript.

Kind regards,

David Paul, Ph.D

Academic Editor

PLOS ONE

Additional Editor Comments:

Based upon comments from two reviewers, the manuscript may be acceptable for publication if the authors can address the concerns expressed by the reviewers. Please address each comment from the reviewers carefully and thoroughly.

Journal Requirements:

When submitting your revision, we need you to address these additional requirements.

1. Please ensure that your manuscript meets PLOS ONE's style requirements, including those for file naming. The PLOS ONE style templates can be found at

https://journals.plos.org/plosone/s/file?id=wjVg/PLOSOne_formatting_sample_main_body.pdf and

https://journals.plos.org/plosone/s/file?id=ba62/PLOSOne_formatting_sample_title_authors_affiliations.pdf

2. Thank you for stating the following in the Financial Disclosure section:

"DB received the funding for this study. This study was partly funded by Lindhés Advokatbyrå and by Södermalm municipally. The funders had no role in study design, data collection and analysis, decision to publish or preparation of the manuscript. "

We note that you received funding from a commercial source: Lindhés Advokatbyrå.

a. Please provide an amended Competing Interests Statement that explicitly states this commercial funder, along with any other relevant declarations relating to employment, consultancy, patents, products in development, marketed products, etc.

Within this Competing Interests Statement, please confirm that this does not alter your adherence to all PLOS ONE policies on sharing data and materials by including the following statement: "This does not alter our adherence to PLOS ONE policies on sharing data and materials.” (as detailed online in our guide for authors http://journals.plos.org/plosone/s/competing-interests).  If there are restrictions on sharing of data and/or materials, please state these. Please note that we cannot proceed with consideration of your article until this information has been declared.

b. Please include your amended Competing Interests Statement within your cover letter. We will change the online submission form on your behalf.

Please know it is PLOS ONE policy for corresponding authors to declare, on behalf of all authors, all potential competing interests for the purposes of transparency. PLOS defines a competing interest as anything that interferes with, or could reasonably be perceived as interfering with, the full and objective presentation, peer review, editorial decision-making, or publication of research or non-research articles submitted to one of the journals. Competing interests can be financial or non-financial, professional, or personal. Competing interests can arise in relationship to an organization or another person. Please follow this link to our website for more details on competing interests: http://journals.plos.org/plosone/s/competing-interests

3. We note that you have indicated that data from this study are available upon request. PLOS only allows data to be available upon request if there are legal or ethical restrictions on sharing data publicly. For information on unacceptable data access restrictions, please see http://journals.plos.org/plosone/s/data-availability#loc-unacceptable-data-access-restrictions.

In your revised cover letter, please address the following prompts:

a) If there are ethical or legal restrictions on sharing a de-identified data set, please explain them in detail (e.g., data contain potentially identifying or sensitive patient information) and who has imposed them (e.g., an ethics committee). Please also provide contact information for a data access committee, ethics committee, or other institutional body to which data requests may be sent.

b) If there are no restrictions, please upload the minimal anonymized data set necessary to replicate your study findings as either Supporting Information files or to a stable, public repository and provide us with the relevant URLs, DOIs, or accession numbers. Please see http://www.bmj.com/content/340/bmj.c181.long for guidelines on how to de-identify and prepare clinical data for publication. For a list of acceptable repositories, please see http://journals.plos.org/plosone/s/data-availability#loc-recommended-repositories.

We will update your Data Availability statement on your behalf to reflect the information you provide.

4. Please include captions for your Supporting Information files at the end of your manuscript, and update any in-text citations to match accordingly. Please see our Supporting Information guidelines for more information: http://journals.plos.org/plosone/s/supporting-information

[Note: HTML markup is below. Please do not edit.]

Reviewers' comments:

Reviewer's Responses to Questions

**Comments to the Author**

1. Is the manuscript technically sound, and do the data support the conclusions?

The manuscript must describe a technically sound piece of scientific research with data that supports the conclusions. Experiments must have been conducted rigorously, with appropriate controls, replication, and sample sizes. The conclusions must be drawn appropriately based on the data presented. 

Reviewer #1: Yes

Reviewer #2: Yes

**********

2. Has the statistical analysis been performed appropriately and rigorously? 

Reviewer #1: Yes

Reviewer #2: Yes

**********

3. Have the authors made all data underlying the findings in their manuscript fully available?

The PLOS Data policy requires authors to make all data underlying the findings described in their manuscript fully available without restriction, with rare exception (please refer to the Data Availability Statement in the manuscript PDF file). The data should be provided as part of the manuscript or its supporting information, or deposited to a public repository. For example, in addition to summary statistics, the data points behind means, medians and variance measures should be available. If there are restrictions on publicly sharing data—e.g. participant privacy or use of data from a third party—those must be specified.

Reviewer #1: Yes

Reviewer #2: Yes

**********

4. Is the manuscript presented in an intelligible fashion and written in standard English?

PLOS ONE does not copyedit accepted manuscripts, so the language in submitted articles must be clear, correct, and unambiguous. Any typographical or grammatical errors should be corrected at revision, so please note any specific errors here.

Reviewer #1: Yes

Reviewer #2: Yes

**********

5. Review Comments to the Author

Please use the space provided to explain your answers to the questions above. You may also include additional comments for the author, including concerns about dual publication, research ethics, or publication ethics. (Please upload your review as an attachment if it exceeds 20,000 characters)

Reviewer #1: This is a cross-sectional data analysis examining the association of policy, playground size, time spent outdoors, and preschool teachers’ physical activity with device-based measurement of preschool-aged children’s physical activity and sedentary time during preschool. This dataset included 369 children, 84 teachers, and 27 preschools in Sweden. The use of device-based measurement of physical activity at both the child and teacher level is a strength of this study. While this work has the potential to support intervention work to promote physical activity and healthy development in young children, there are a few concerns that should be addressed first.

Major comments:

1) The introduction would be strengthened by the inclusion and restructuring of paragraphs based on theory. For example, the social ecological model would lend itself to this work nicely. Additionally, a paragraph of policies supporting physical activity in preschool is needed. Restructuring the flow of the paragraphs would help the reader with the overall focus of this paper.

2) While the use of device-based physical activity measurement is a strength of this paper, two major concerns arise with data management.

a. Accelerometer best practices are different by age group (starting line 148). For preschoolers, 5-15 second epoch lengths are typically used due to the sporadic nature of activity within this age group. In children, longer epoch lengths (e.g. 60-sec) result in less resolution and lower minutes of higher intensity activity (Migueles et al., 2017) and sedentary time has been found to be reclassified as LPA with larger epoch lengths (Banda et al., 2016). This may be why this sample had a lower end of % time in sedentary and higher end of % spent in LPA. Please address why 60-second epoch length was utilized for the preschool-aged sample.

b. It is unclear what the typical hours children attend preschool (line 153) and how the accelerometer reduction protocol reflects “all preschool hours”. The results section (line 195) is the first mention of removing participants that had less than 10 hours/day of wear time during preschool hours. 10 hours/day is typically used for whole day accelerometer wear. Further, line 199 reports children “spent 475 minutes (7.9h) in preschool per day”. This seems contradictory to the exclusion criteria of children who had less than “… 10 hours/day of accelerometer measures during preschool hours…” (line 195-196), which would equate to a minimum of 600 minutes. Therefore, it would be beneficial to describe the overall operational structure of the preschools.

3) While the authors use a statistical approach that allows for the nested data, it is unclear why teachers’ physical activity was aggregated at preschool-level (ultimately lowering total n and variability) and not clustered within classroom. Please explain the rationale behind not utilizing a 3-level approach – nesting students within teachers and teachers within schools. Do teachers interact with all children at the center? If not, please also include the average number of children per class.

Minor comments:

1) In the introduction, please define preschool age (line 71, line 74). 1-5 years mentioned in into (line 78), yet study design is 3-5 years (line 115)

2) EPAO-SR is a large survey, which subscales/items of the EPAO-SR were used? (line 127)

3) Light PA (line 156) has been previously defined (line 73)

4) What is the cluster effect at the preschool-level, i.e. how much of the variance in the physical activity outcome data were attributed to school?

5) Were their correlations between the independent variables? Are schools with larger playground areas more likely to spend more time outdoors? This may explain some of the findings for playground area for joint model.

6) Line 239 – unclear why only Fig 3/ steps is discussed as dose-response for independent models when MVPA and LPA show similar findings.

7) Results have no discussion of sedentary time findings.

8) A limitation not noted is the dichotomous PA policy score. The EPAO-SR includes several policies that are specifically related to this study’s interest such as outdoors and adult-led activities. Reducing this to dichotomous variables may impact results as specific PA-related policies support meeting best practices at varying odds/prevalence (Dooley et al., 2020 Examining physical activity policies to practice implementation; Erinosho et al., 2016 Impact of policies on physical activity and screen time practices in 50 child-care centers in North Carolina; Sisson et al., 2012 Assessment of food, nutrition, and physical activity practices in Oklahoma child-care centers)

9) Would be helpful to include total accelerometer wear time and chi-square/t-test p-values in Table 1.

10) Would be helpful to include p-values for differences across groups for Table S4. Additionally, if possible, it would be beneficial to include the number of children excluded based on “incomplete accelerometer data”, “missing anthropometrics” and/or “missing data on time spent in- and outdoors” within the text for lines 276-278 or earlier in lines 196-197. Could there be center-level characteristics that led to exclusions – were excluded children random across centers or clustered in a few centers? Seems like children attending centers that do not have a formalized polices and smaller playground areas were more likely to be excluded.

Reviewer #2: Review of Manuscript for PLOS ONE

Thank you for the opportunity to review the paper entitled: ‘Preschool environment and preschool teacher’s physical activity and their association with children’s activity levels at preschool’. The manuscript provides detail of a study assessing the extent to which the preschool environment is associated with children’s physical activity, steps and sedentary time. This paper communicates original research and the findings provide some more insight into the activity levels of pre-schoolers with a novel inclusion of their teachers. I have reviewed the manuscript in its entirety. The paper is well-written and worthy of acceptance without revisions. There are two minor edits you may wish to attend to below. Congratulations.

Page 4 line 87- suggest changing the following wording: Studies on associations to Studies investigating associations

Page 15 line 311- add a word in the sentence: a review on the promotion of PA

**********

6. PLOS authors have the option to publish the peer review history of their article (what does this mean?). If published, this will include your full peer review and any attached files.

If you choose “no”, your identity will remain anonymous but your review may still be made public.

**Do you want your identity to be public for this peer review?** For information about this choice, including consent withdrawal, please see our Privacy Policy.

Reviewer #1: No

Reviewer #2: No

[NOTE: If reviewer comments were submitted as an attachment file, they will be attached to this email and accessible via the submission site. Please log into your account, locate the manuscript record, and check for the action link "View Attachments". If this link does not appear, there are no attachment files.]

While revising your submission, please upload your figure files to the Preflight Analysis and Conversion Engine (PACE) digital diagnostic tool, https://pacev2.apexcovantage.com/. PACE helps ensure that figures meet PLOS requirements. To use PACE, you must first register as a user. Registration is free. Then, login and navigate to the UPLOAD tab, where you will find detailed instructions on how to use the tool. If you encounter any issues or have any questions when using PACE, please email PLOS at figures@plos.org. Please note that Supporting Information files do not need this step.

# Author comment: Author response to Decision Letter 0

**DOI:** 10.1371/journal.pone.0239838.r002

30 Jun 2020

Point by point response to editor and reviewers 

Additional Editor Comments:

Based upon comments from two reviewers, the manuscript may be acceptable for publication if the authors can address the concerns expressed by the reviewers. Please address each comment from the reviewers carefully and thoroughly.

Answer: We are very pleased to hear that the manuscript is under consideration. We recognize that all the comments and the process of peer review have greatly improved the clarity of our manuscript. We have addressed all comments by editor and reviewers point by point to the best of our ability. 

Journal Requirements:

When submitting your revision, we need you to address these additional requirements.

1. Please ensure that your manuscript meets PLOS ONE's style requirements, including those for file naming. The PLOS ONE style templates can be found at

https://journals.plos.org/plosone/s/file?id=wjVg/PLOSOne_formatting_sample_main_body.pdf and

https://journals.plos.org/plosone/s/file?id=ba62/PLOSOne_formatting_sample_title_authors_affiliations.pdf

Answer: Thank you for your comment and we concur that meeting the style requirements is important for the standardized and concise presentation of the manuscript. We apologize for the formation that did not meet the style requirements and have corrected them in the revised manuscript. We have realized and corrected the following 7 points: 

1) Affiliation format: we have now added this missing city information in second and third affiliations.

(page 1, line 11): “2Department of Global Public Health, Karolinska Institutet, Solna, Sweden”

(page 1, line 13): “3Department of Biosciences and Nutrition, Karolinska Institutet, Solna, Sweden”

2) The figure legends have been moved from a new line following the figure tile to directly following the figure tile. 

(page 13, line 277): “Fig 2. Association between predictors and children’s MVPA during preschool time. Both models are adjusted for age, sex and BMI category Abbreviations: PA = physical activity, MVPA = moderate to vigorous physical activity, 

Q1-4 = quartile 1-4, BMI = body mass index”

(page 13, line 282): “Fig 3. Association between predictors and children’s LPA during preschool time. Both models are adjusted for age, sex and BMI category Abbreviations: PA = physical activity, LPA = light physical activity, Q1-4 = quartile 1-4, BMI = body mass index”

(page 13, line 286): “Fig 4. Association between predictors and children’s steps during preschool time. Both models are adjusted for age, sex and BMI category

Abbreviations: Q1-4 = quartile 1-4, BMI = body mass index”

(page 14, line 290): “Fig 5. Association between predictors and children’s ST during preschool time. Both models are adjusted for age, sex and BMI category

Abbreviations: Q1-4 = quartile 1-4, BMI = body mass index”

(page 15, line 324): “Fig 6. Association between preschool teachers’ and children’s PA and ST during preschool time. Abbreviations: PA = physical activity, MVPA = moderate to vigorous physical activity, LPA=light physical activity, ST=sedentary time”

3) Formats of multiple citation of figures have been corrected, for example, from “Fig 1-3” to “Figs 1-3”. 

(page 13, line 269): “When formalized preschool policy was analyzed as the only predictor in the independent models, the effect of formalized preschool PA policy was hardly manifested (Figs 2-5, independent model).”

(page 13, line 270): “However, when all predictors were analyzed simultaneously (Figs 2-5, joint model), i.e. the direct association between each predictor and outcome,” 

(page 14, line 269): “As is shown in Figs 2-4, the independent model shows dose-response association between preschool playground area and children’s PA and steps.”

4) Title of Table 1 has been moved from under the table to above the table. 

5) All citation of references in the manuscript has been changed, for example, from “(1)” to “[1]”.

6) Supporting information captions have been added to the end of the revised manuscript. 

7) Size of the font has been changed according to requirement. 

2. Thank you for stating the following in the Financial Disclosure section:

"DB received the funding for this study. This study was partly funded by Lindhés Advokatbyrå and by Södermalm municipally. The funders had no role in study design, data collection and analysis, decision to publish or preparation of the manuscript. "

We note that you received funding from a commercial source: Lindhés Advokatbyrå.

a. Please provide an amended Competing Interests Statement that explicitly states this commercial funder, along with any other relevant declarations relating to employment, consultancy, patents, products in development, marketed products, etc.

Within this Competing Interests Statement, please confirm that this does not alter your adherence to all PLOS ONE policies on sharing data and materials by including the following statement: "This does not alter our adherence to PLOS ONE policies on sharing data and materials.” (as detailed online in our guide for authors http://journals.plos.org/plosone/s/competing-interests). If there are restrictions on sharing of data and/or materials, please state these. Please note that we cannot proceed with consideration of your article until this information has been declared.

Answer: We appreciate your comments on competing interest as we concur that this is important for the objective presentation of the research. We have clarified about the declaration regarding Lindhés Advokatbyrå in the cover letter for revision as “Lindhés Advokatbyrå has no competing interest in terms of its ownership of stocks, employment or consultancy, board membership, patent applications (pending or actual), research grants, travel grants or gifts. Receiving funding from Lindhés Advokatbyrå does not alter our adherence to all PLOS ONE policies on sharing data and materials. However, our dataset cannot be made publicly available due to both legal and ethical considerations. All healthcare data are considered as sensitive data in Sweden; therefore, the sharing of our dataset is legally restricted by Regional Ethical Review Board. In addition, restriction of public sharing of dataset was included in the informed consent document signed by the participant therefore sharing the dataset would be unethical. However, the dataset can be made available if the request for dataset meet the access criteria from The Regional Ethical Board (contact website: https://etikprovningsmyndigheten.se/).”

 b. Please include your amended Competing Interests Statement within your cover letter. We will change the online submission form on your behalf.

Please know it is PLOS ONE policy for corresponding authors to declare, on behalf of all authors, all potential competing interests for the purposes of transparency. PLOS defines a competing interest as anything that interferes with, or could reasonably be perceived as interfering with, the full and objective presentation, peer review, editorial decision-making, or publication of research or non-research articles submitted to one of the journals. Competing interests can be financial or non-financial, professional, or personal. Competing interests can arise in relationship to an organization or another person. Please follow this link to our website for more details on competing interests: http://journals.plos.org/plosone/s/competing-interests

Answer: Thank you for your comment and the amended Competing Interests Statement has been added to cover letter as stated above. 

3. We note that you have indicated that data from this study are available upon request. PLOS only allows data to be available upon request if there are legal or ethical restrictions on sharing data publicly. For information on unacceptable data access restrictions, please see http://journals.plos.org/plosone/s/data-availability#loc-unacceptable-data-access-restrictions.

In your revised cover letter, please address the following prompts:

a) If there are ethical or legal restrictions on sharing a de-identified data set, please explain them in detail (e.g., data contain potentially identifying or sensitive patient information) and who has imposed them (e.g., an ethics committee). Please also provide contact information for a data access committee, ethics committee, or other institutional body to which data requests may be sent.

Answer: Thank you for your comment and we agree that publicly available datasets enhance the transparency of research. However, our can data can not be made publicly available due both legal and ethical issues. Our data is considered healthcare sensitive data therefore have legal restriction for sharing from Regional Ethical Review Board. Further, the restriction of sharing data has been informed to the participations and they consented their participation upon this restriction of data sharing. Therefore, sharing our dataset would also be unethical. However, reasonable request for the dataset may be granted from the Regional Ethical Review Board (https://etikprovningsmyndigheten.se/) if the access criteria are met. 

We have explained about this in the cover letter as: “All healthcare data are considered as sensitive data in Sweden; therefore, the sharing of our dataset is legally restricted by Regional Ethical Review Board. In addition, restriction of public sharing of dataset was included in the informed consent document signed by the participant therefore sharing the dataset would be unethical. However, the dataset can be made available if the request for dataset meet the access criteria from The Regional Ethical Board (contact website: https://etikprovningsmyndigheten.se/).”

b) If there are no restrictions, please upload the minimal anonymized data set necessary to replicate your study findings as either Supporting Information files or to a stable, public repository and provide us with the relevant URLs, DOIs, or accession numbers. Please see http://www.bmj.com/content/340/bmj.c181.long for guidelines on how to de-identify and prepare clinical data for publication. For a list of acceptable repositories, please see http://journals.plos.org/plosone/s/data-availability#loc-recommended-repositories.

We will update your Data Availability statement on your behalf to reflect the information you provide.

Answer: There is restriction as stated above, therefore we cannot upload our dataset. 

4. Please include captions for your Supporting Information files at the end of your manuscript, and update any in-text citations to match accordingly. Please see our Supporting Information guidelines for more information: http://journals.plos.org/plosone/s/supporting-information

Answer: Thank you for your comment and we have now included the supporting information at the end of the revised manuscript as following and updated the matching in-text citation. 

Supporting Information

S1 Table. Cross tabulation of teachers' PA and children' s PA High and low are classified by the median of the respective teacher PA variable Abbreviations: PA = physical activity, MVPA = moderate to vigorous physical activity, LPA = light physical activity, ST = sedentary time

S2 Table. Associations between predictors and physical activity indicators during preschool time (n=369) Model 1 = crude model each predictor independently, Model 2 = Model 1 adjusted for age, sex, BMI category Model 3= all predictors jointly, Model4 = Model 3 adjusted for age, sex, BMI category Abbreviations: PA = physical activity, BMI = body mass index, MVPA = moderate to vigorous physical activity, LPA = light physical activity, ST = sedentary time, Q1-4 = quartile 1-4 Reference level: Formalized PA policy = No, Playground area = ≤200 m2, Time spend outdoors = Q1

S3 Table Preschool-level cluster effect (Intra class correlation) in each Linear Mixed Model Model 1 = crude model each predictor independently, Model 2 = Model 1 adjusted for age, sex, BMI category Model 3= all predictors jointly, Model4 = Model 3 adjusted for age, sex, age, BMI category Abbreviations: MVPA = moderate to vigorous physical activity, LPA = light physical activity, ST = sedentary time Reference level: Formalized PA policy = No, Playground area = ≤200 m2, Time spend outdoors = Q1

S4 Table. Associations between predictors and physical activity indicators during preschool time (n=337) Model 1 = crude model each predictor independently, Model 2 = Model 1 adjusted for age, sex, BMI category and parental education Model 3= all predictors jointly, Model4 = Model 3 adjusted for age, sex, age, BMI category and parental education Abbreviations: PA = physical activity, BMI = body mass index, MVPA = moderate to vigorous physical activity, LPA = light physical activity, ST = sedentary time, Q1-4 = quartile 1-4 Reference level: Formalized PA policy = No, Playground area = ≤200 m2, Time spend outdoors = Q1

S5 Table. Comparison of descriptive characteristics between analytical dataset and excluded observations PA = physical activity, BMI = body mass index, MVPA = moderate to vigorous physical activity, LPA = light physical activity, ST = sedentary time, Q1-4 = quartile 1-4

S6. Table Policy content in seven preschool reported formalized policy

For format in in-text citation: (page 15, line 330)

In sensitivity analyses, we estimated the intra class correlation of each model shown in S2 Table. We observed small to modest ICC for all models (and all outcomes); there was a small ICC for MVPA, and somewhat higher for Steps, LPA and ST (S3 Table). In addition, we ran all models included in S2 Table with additional adjustment made for parental education in a subset population of children (n=337) (S4 Table). S4 Table shows that none of the estimates are affected, to any substantial extent, by the introduction of parental education as a covariate. Finally, we ran descriptive analyses comparing children in the analytical sample (n=369) with children with incomplete accelerometer data or missing data on anthropometrics and or missing data on time spent in- and outdoors (n=35) (S5 Table). As shown in S5 Table, children excluded from analyses, due to missing data, had a higher prevalence of obesity (33% vs. 2%) and lower PA level. The excluded sample were generally distributed across the preschools except for that one preschool contributed to 11 (31.4%) exclusions due to loss of sub-report document for in/out report with information on preschool time and time spent outdoors. This preschool had the smallest preschool playground and did not have a formalized policy. 

Reviewers’ comments

Reviewer #1: This is a cross-sectional data analysis examining the association of policy, playground size, time spent outdoors, and preschool teachers’ physical activity with device-based measurement of preschool-aged children’s physical activity and sedentary time during preschool. This dataset included 369 children, 84 teachers, and 27 preschools in Sweden. The use of device-based measurement of physical activity at both the child and teacher level is a strength of this study. While this work has the potential to support intervention work to promote physical activity and healthy development in young children, there are a few concerns that should be addressed first.

Answer: Thank you for your acknowledgment of our study, we are glad to hear that you agree that the provision of objective data on physical activity in the understudied preschool population with inclusion of objective data on teachers is the strength and novelty of our study. We strived to contribute to the evidence base for environmental/organizational intervention in early childhood education and care setting given the knowledge gaps. Thank you for providing constructive feedbacks to our paper and we believe we have improved the clarity of our manuscript by addressing the comments. The comments are addressed point by point below. 

Major comments:

1) The introduction would be strengthened by the inclusion and restructuring of paragraphs based on theory. For example, the social ecological model would lend itself to this work nicely. Additionally, a paragraph of policies supporting physical activity in preschool is needed. Restructuring the flow of the paragraphs would help the reader with the overall focus of this paper.

Answer: Thank you for your comment and suggestion, we agree that a theory base would strengthen our introduction and more information on the preschool physical activity policies would highlight the focus of the paper. According to the review by Finch et al, the use of theory is associated with greater intervention effect in physical activity interventions in childcare setting (Finch, Jones et al. 2016). Although not specifically stated in our manuscript, our attempt to understand the context that promote higher physical activity was stimulated by the social ecological model. The social ecological model suggests that there are individual level, interpersonal level, organizational level and physical environment level determinants of health behavior (Bronfenbrenner 1979). We concur that the provision of this model builds a clear structure in the factors that we investigate in our research; Playground size on the physical environment level, formalized policy and time spent outdoors on the organizational level and teachers’ physical activity on the interpersonal level. 

In addition to the provision of theory base, we have summarized the unrevised and revised text flow to illustrate the change in structure. Knowledge gaps have also been further highlighted in the introduction to help the readers to grasp the overall focus of the paper.

The unrevised text flow was as follows: Benefits of physical activity and the need of PA promoting intervention � Importance of environmental strategy and preschool being the potential environment for intervention� Modifiable environmental factors in preschools � Role of preschool teachers � research gap and the aim of this study. 

The revised text flow is now as follows: Benefits of physical activity and the need of PA promoting intervention � Rationale for inclusion of theory and introduction of theory, preschool being the potential place for intervention � potential modifiable factors with focus on physical environmental level �Organizational level factors such as policy and time spent outdoors �interpersonal level factor in terms of role of preschool teachers (preschool teachers’ PA level) � aim of the study 

Since we have reorganized the introduction, the whole section is shown here with changed parts highlighted for your convenience of reading. 

Introduction

Total physical activity (PA), moderate to vigorous PA (MVPA) (Carson, Lee et al. 2017) and steps per day (Tudor-Locke, Craig et al. 2011) are positively associated with multiple health indicators in young children, while more conflicting findings have been reported for sedentary time (ST) (Cliff, Hesketh et al. 2016). Furthermore, several studies have shown that physically active children tend to remain more physically active across their lifespan (Jones, Hinkley et al. 2013). Despite the many known benefits of PA, children are in general physically inactive (Bornstein, Beets et al. 2011). A review on preschoolers’ physical activity level based on objective measure has shown highly variable results that preschool children spend 2%–41% of their day in MVPA, 4%–33% in light PA (LPA), and 34%–94% sedentary (Hnatiuk, Salmon et al. 2014). Moreover, Swedish data with objectively measured PA and ST, show that preschoolers’ levels of PA are low (Berglind, Hansson et al. 2016, Berglind and Tynelius 2017). Hence, effective strategies informed by objective data are urgently required to promote child PA. 

A theory base is suggested to be beneficial for the effectiveness of PA promoting strategies (Finch, Jones et al. 2016), therefore the social ecological model was employed as framework to understand systematically what factors may enable effective PA promotion in preschool children (Bronfenbrenner 1979). According to the social ecological model, there are different levels of determinants of health behaviors such as physical activity namely individual level, interpersonal level, organizational level and physical environment level (Bronfenbrenner 1979). Environmental intervention in preschool which lay emphasize on non-individual level determinants of PA, can potentially be an effective PA promotion strategy also addressing sustainability and equity, but the evidence-base is scarce (Mehtälä, Sääkslahti et al. 2014). In Sweden, approximately 92% of all children 1-5 years of age are attending preschools, regardless of their parents’ socioeconomic status (Pearce, Page et al. 2014). Furthermore, approximately 50% of children’s daily MVPA is accumulated during preschool hours (Berglind and Tynelius 2017). Therefore, the preschool environment presents an ideal arena to promote early development of healthy PA and ST behaviors (Gordon, Tucker et al. 2013). 

Potential modifiable characteristics for preschool includes physical environment, policy and time spent outdoors on the organizational level and teachers’ PA on the interpersonal level, but the evidence supporting effectiveness is preliminary. In terms of interventions to modify physical environment, studies have shown that structural environmental factors such as playground size, play equipment accessibility and design of the preschool playground may be of importance for children’s PA during preschool hours (Broekhuizen, Scholten et al. 2014, Hinkley, Carson et al. 2015). However, consensus on playground size is hampered by the lack of evidence, application of objective measure on PA, and the difficulty in studying this issue with randomized experimental design (Broekhuizen, Scholten et al. 2014). Although physical environment level interventions address all children in the environment with potentially promising sustainability, they are seldom practical due to the requirement on resources especially on large scale (Swerissen and Crisp 2004). 

Modifying organizational factors, such as policy and time spent outdoors, is less resource-dependent but may be effective provided adequate evidence base. Having a PA policy in preschool is suggested to be beneficial for preschooler’s PA but objective measure on PA is lacking (Hinkley, Carson et al. 2015, Byrd-Williams, Dooley et al. 2019, Dooley, Thi et al. 2020). Conflicting results have been demonstrated by the few existing studies with accelerometer data. While Dowda et al found more MVPA in PA promoting preschools where policy is one of the components (Dowda, Brown et al. 2009), Erinosho et al showed a negative association between PA policy and accelerometer measured PA level in preschool children (Erinosho, Hales et al. 2016). More research with objective PA data is needed to determine the association between policy and PA levels in preschool children. Similarly, studies investigating associations between time spent outdoors at preschool and children’s levels of PA using objective measures on PA are also scarce. Only a few existing studies indicate that the amount of time preschool children spend outdoors is positively associated with their levels of PA and negatively associated with ST (Boldemann, Blennow et al. 2006, Tandon, Saelens et al. 2018). A recent randomized controlled trial showed that scheduling both shorter more frequent and longer outdoor sessions during preschool hours significantly increased preschool children’s MVPA (Razak, Yoong et al. 2018). Thus, increasing time spent outdoors during preschool hours may, in addition to policies, be an effective strategy to promote healthy PA among preschool children. However, more studies are warranted to further explore the potential of organizational level factors. 

Interpersonal level factor such as preschool teacher’s attitude, initiative, and participation in physical activities along with children, may play an important role in promoting preschool children’s PA (Mikkelsen 2011). However, there is a lack of studies among the preschool population. Only one Norwegian study has used objectively measured PA in both preschool teachers’ and children and found a small, but statistically significant association, between preschool teachers’ and preschool children’s levels of PA during preschool hours (Fossdal, Kippe et al. 2018). More studies with objective measure on both preschool teachers and children are imperative to confirm the potential association between preschooler’s PA and preschool teachers’ PA. 

To address these knowledge gaps, the aim of the current study is to assess to what extent the physical preschool environment, formalized PA policies, time spent outdoors and preschool teachers’ levels of PA were associated with children’s objectively assessed PA, steps and ST during preschool hours to deepen knowledge in informing strategy development for child PA promotion. 

References:

Berglind, D., L. Hansson, P. Tynelius and F. Rasmussen (2016). "Levels and Patterns of Objectively Measured Physical Activity and Sedentary Time in Four-Year-Old Swedish Children." J Phys Act Health: 1-23.

Berglind, D. and P. Tynelius (2017). "Objectively measured physical activity patterns, sedentary time and parent-reported screen-time across the day in four-year-old Swedish children." BMC Public Health 18(1): 69.

Boldemann, C., M. Blennow, H. Dal, F. Martensson, A. Raustorp, K. Yuen and U. Wester (2006). "Impact of preschool environment upon children's physical activity and sun exposure." Preventive Medicine 42(4): 301-308.

Bornstein, D. B., M. W. Beets, W. Byun and K. McIver (2011). "Accelerometer-derived physical activity levels of preschoolers: a meta-analysis." Journal of Science and Medicine in Sport 14(6): 504-511.

Broekhuizen, K., A.-M. Scholten and S. I. de Vries (2014). "The value of (pre)school playgrounds for children’s physical activity level: a systematic review." International Journal of Behavioral Nutrition and Physical Activity 11(1): 59.

Bronfenbrenner, U. (1979). The ecology of human development, Harvard university press.

Byrd-Williams, C. E., E. E. Dooley, C. A. Thi, C. Browning and D. M. Hoelscher (2019). "Physical activity, screen time, and outdoor learning environment practices and policy implementation: a cross sectional study of Texas child care centers." BMC Public Health 19(1): 274.

Carson, V., E. Y. Lee, L. Hewitt, C. Jennings, S. Hunter, N. Kuzik, J. A. Stearns, S. P. Unrau, V. J. Poitras, C. Gray, K. B. Adamo, I. Janssen, A. D. Okely, J. C. Spence, B. W. Timmons, M. Sampson and M. S. Tremblay (2017). "Systematic review of the relationships between physical activity and health indicators in the early years (0-4 years)." BMC Public Health 17(Suppl 5): 854.

Cliff, D. P., K. D. Hesketh, S. A. Vella, T. Hinkley, M. D. Tsiros, N. D. Ridgers, A. Carver, J. Veitch, A. M. Parrish, L. L. Hardy, R. C. Plotnikoff, A. D. Okely, J. Salmon and D. R. Lubans (2016). "Objectively measured sedentary behaviour and health and development in children and adolescents: systematic review and meta-analysis." Obes Rev 17(4): 330-344.

Dooley, E. E., C. A. Thi, C. Browning, D. M. Hoelscher and C. E. Byrd-Williams (2020). "Examining physical activity policies to practice implementation: Results from the Texas Early Childhood Physical Activity Survey in non-Head Start childcare centers." Prev Med Rep 17: 101019.

Dowda, M., W. H. Brown, K. L. McIver, K. A. Pfeiffer, J. R. O'Neill, C. L. Addy and R. R. Pate (2009). "Policies and characteristics of the preschool environment and physical activity of young children." Pediatrics 123(2): e261-266.

Erinosho, T., D. Hales, A. Vaughn, S. Mazzucca and D. S. Ward (2016). "Impact of Policies on Physical Activity and Screen Time Practices in 50 Child-Care Centers in North Carolina." J Phys Act Health 13(1): 59-66.

Finch, M., J. Jones, S. Yoong, J. Wiggers and L. Wolfenden (2016). "Effectiveness of centre-based childcare interventions in increasing child physical activity: a systematic review and meta-analysis for policymakers and practitioners." Obes Rev 17(5): 412-428.

Fossdal, T. S., K. Kippe, B. H. Handegard and P. Lagestad (2018). ""Oh oobe doo, I wanna be like you" associations between physical activity of preschool staff and preschool children." Plos One 13(11).

Gordon, E. S., P. Tucker, S. M. Burke and A. V. Carron (2013). "Effectiveness of physical activity interventions for preschoolers: a meta-analysis." Res Q Exerc Sport 84(3): 287-294.

Hinkley, T., V. Carson and K. D. Hesketh (2015). "Physical environments, policies and practices for physical activity and screen-based sedentary behaviour among preschoolers within child care centres in Melbourne, Australia and Kingston, Canada." Child: Care, Health and Development 41(1): 132-138.

Hnatiuk, J. A., J. Salmon, T. Hinkley, A. D. Okely and S. Trost (2014). "A review of preschool children's physical activity and sedentary time using objective measures." Am J Prev Med 47(4): 487-497.

Jones, R. A., T. Hinkley, A. D. Okely and J. Salmon (2013). "Tracking Physical Activity and Sedentary Behavior in Childhood: A Systematic Review." American Journal of Preventive Medicine 44(6): 651-658.

Mehtälä, M. A. K., A. K. Sääkslahti, M. E. Inkinen and M. E. H. Poskiparta (2014). "A socio-ecological approach to physical activity interventions in childcare: a systematic review." International Journal of Behavioral Nutrition and Physical Activity 11(1): 22.

Mikkelsen, B. E. (2011). "Associations between pedagogues attitudes, praxis and policy in relation to physical activity of children in kindergarten - results from a cross sectional study of health behaviour amongst Danish pre-school children." International Journal of Pediatric Obesity 6: 12-15.

Pearce, M., A. S. Page, T. P. Griffin and A. R. Cooper (2014). "Who children spend time with after school: associations with objectively recorded indoor and outdoor physical activity." Int J Behav Nutr Phys Act 11(1): 45.

Razak, L. A., S. L. Yoong, J. Wiggers, P. J. Morgan, J. Jones, M. Finch, R. Sutherland, C. Lecathelnais, K. Gillham, T. Clinton-McHarg and L. Wolfenden (2018). "Impact of scheduling multiple outdoor free-play periods in childcare on child moderate-to-vigorous physical activity: a cluster randomised trial." International Journal of Behavioral Nutrition and Physical Activity 15.

Swerissen, H. and B. R. Crisp (2004). "The sustainability of health promotion interventions for different levels of social organization." Health promotion international 19(1): 123-130.

Tandon, P. S., B. E. Saelens, C. Zhou and D. A. Christakis (2018). "A Comparison of Preschoolers' Physical Activity Indoors versus Outdoors at Child Care." International Journal of Environmental Research and Public Health 15(11).

Tudor-Locke, C., C. L. Craig, M. W. Beets, S. Belton, G. M. Cardon, S. Duncan, Y. Hatano, D. R. Lubans, T. S. Olds, A. Raustorp, D. A. Rowe, J. C. Spence, S. Tanaka and S. N. Blair (2011). "How Many Steps/Day are Enough? for Children and Adolescents." International Journal of Behavioral Nutrition and Physical Activity 8.

2) While the use of device-based physical activity measurement is a strength of this paper, two major concerns arise with data management.

a. Accelerometer best practices are different by age group (starting line 148). For preschoolers, 5-15 second epoch lengths are typically used due to the sporadic nature of activity within this age group. In children, longer epoch lengths (e.g. 60-sec) result in less resolution and lower minutes of higher intensity activity (Migueles et al., 2017) and sedentary time has been found to be reclassified as LPA with larger epoch lengths (Banda et al., 2016). This may be why this sample had a lower end of % time in sedentary and higher end of % spent in LPA. Please address why 60-second epoch length was utilized for the preschool-aged sample.

Answer: Thank you for your comment and we concur that shorter epoch-length have been suggested to be more suitable for young children. However, we used cut points to classify physical activity intensity level according to Butte et al (Butte, Wong et al. 2014) that developed these cut points with 60 second epoch length. The Bette et al cut points have been developed especially for 4-year-old children with Actigraph GT3X+ in free-living setting which suits the target population and condition of our study (Butte, Wong et al. 2014). Generally, we are in favor of using accelerometer configurations which are as proximal to a relevant calibration study as possible - both to ensure validity and to enhance comparability across studies.

Further, cut-point scaling can be conducted to enable a shorter epoch length that may suit young children’ s physical activity pattern. For example, the MVPA cut point of 3908 counts per minute can be scaled to 977 counts per 15 seconds to enable us to use a shorter epoch length in our study. However, studies show that cut point scaling would affect the estimation and are not recommend in both adult (Mueller, Chimenti et al.) and adolescents (Hibbing, Bassett et al. 2020). We expect similar findings in preschool children; the best estimation of physical activity levels is made when the conditions of the validated calibration study are strictly followed. Therefore, we chose to strictly follow the calibration epoch of 60 seconds rather than tailor it to suit a shorter epoch. 

However, as we concur with the reviewer that there are arguments for shorter epoch length and we see how readers may share this concern, we have added the rationale for using 60 second epoch in method section (page 8, line 169): “A 60 s epoch length was used in analysis according to the epoch setting in the validation study that developed these cut-offs (Butte, Wong et al. 2014).”

Further, as we concur with the ongoing discussion, we have added a description of our rationale for the use of 60s epoch in preschool children in strength and limitation section (page 20, line 447) as “In addition, a 60s epoch was adopted in accelerometer data analysis while a shorter epoch length has been suggested to suit the young children’s sporadic moving nature (Migueles, Cadenas-Sanchez et al. 2017). However, studies also suggest that scaling the cut-offs to suit a different epoch may be problematic (Hibbing, Bassett et al. 2020). The accuracy of cut-offs to classify PA level may be optimal when they are applied under the same epoch setting as the calibration setting that developed these cut-offs. (Hibbing, Bassett et al. 2020, Marissa Hope, Ruth et al. 2020). Therefore, accelerometer data was analyzed in 60 s epoch strictly following the epoch length used in the validation study (Butte, Wong et al. 2014). ”

References: 

Butte, N. F., W. W. Wong, J. S. Lee, A. L. Adolph, M. R. Puyau and I. F. Zakeri (2014). "Prediction of energy expenditure and physical activity in preschoolers." Med Sci Sports Exerc 46(6): 1216-1226.

Hibbing, P. R., D. R. Bassett and S. E. Crouter (2020). "Modifying Accelerometer Cut-Points Affects Criterion Validity in Simulated Free-Living for Adolescents and Adults." Res Q Exerc Sport: 1-11.

Marissa Hope, M., C. Ruth, M. Shannon and A. F.-L. Laura (2020). "Accelerometry Analysis Options Produce Large Differences in Lifestyle Physical Activity Measurement." Physiological Measurement.

Migueles, J. H., C. Cadenas-Sanchez, U. Ekelund, C. Delisle Nystrom, J. Mora-Gonzalez, M. Lof, I. Labayen, J. R. Ruiz and F. B. Ortega (2017). "Accelerometer Data Collection and Processing Criteria to Assess Physical Activity and Other Outcomes: A Systematic Review and Practical Considerations." Sports Med.

Mueller, M. A.-O., R. Chimenti, S. Merkle and L. A.-O. Frey-Law "Accelerometry Analysis Options Produce Large Differences in Lifestyle Physical Activity Measurement. LID - 10.1088/1361-6579/ab94d4 [doi]." (1361-6579 (Electronic)).

Tudor-Locke, C., T. V. Barreira and J. M. Schuna (2015). "Comparison of Step Outputs for Waist and Wrist Accelerometer Attachment Sites." Medicine and Science in Sports and Exercise 47(4): 839-842.

b. It is unclear what the typical hours children attend preschool (line 153) and how the accelerometer reduction protocol reflects “all preschool hours”. The results section (line 195) is the first mention of removing participants that had less than 10 hours/day of wear time during preschool hours. 10 hours/day is typically used for whole day accelerometer wear. Further, line 199 reports children “spent 475 minutes (7.9h) in preschool per day”. This seems contradictory to the exclusion criteria of children who had less than “… 10 hours/day of accelerometer measures during preschool hours…” (line 195-196), which would equate to a minimum of 600 minutes. Therefore, it would be beneficial to describe the overall operational structure of the preschools.

Answer: Thank you for your feedback that helped us realize that the way we presented the wear time and preschool hours causes confusion. Children wore the accelerometer the whole day as is stated (unrevised manuscript page 6 line 149) “children and preschool teachers were instructed to wear the accelerometer, at the right hip, all waking hours for 7 consecutive days.”. However, what was considered valid accelerometer data was having at least 3 days, more than 10h/day wear time. This has been misstated (unrevised manuscript page 7, line 152) as “at least 3 days during all preschool hours.” We have corrected this in revised manuscript (page 8, line 191) as “Accelerometer data were considered valid if the child wore the accelerometer for at least 3 days, 10 hours/day.”

To clarify the exclusion of participants, we first removed participants with less than 3 days, 10 hours/day wear time. In participants with valid whole day accelerometer data, the time-stamped accelerometer data was matched with the recorded preschool time data to further extract PA during preschool time because the focus of this study is on the association between children’s physical activity levels and preschool factors. We apologize for the incorrect statement of removing participants who had less than 10 hours/day of wear time during preschool time (unrevised manuscript 195) where in fact we removed participants wo had less than 10 hours/day of wear time per day. 

We have further illustrated the accelerometer data exclusion and extraction process in detail with a flowchart of participants and corrected the misstated exclusion of less than 10h of wear time during preschool time in the results (Page 11, line 246) as “Fig 1 demonstrates the derivation of the analytical dataset. In total, 404 children and 92 preschool teachers from 27 preschools participated in the current study. First, 10 children and 6 teachers were excluded because they had less than 3 days or 10 hours/day of accelerometer data. Second, 25 children and 2 teachers were excluded due to missing recorded preschool hours, as such information were required to determine PA during preschool time. Thus, the final analytical sample comprised 369 children and 84 preschool teachers.”

In addition, we have clarified preschool operation and documentation of preschool hours in the manuscript (Page 9, line 202, in the end of Physical activity and sedentary time, methods section) as “ After the validation and classification of PA level for whole day PA accelerometer measure, the time-stamped accelerometer data was further matched with preschool time information to extract PA during preschool time due to the focus of the preschool factors in this study. In Sweden, preschool hours vary to fit parents’ working schedule, but most preschools are open from 7:00 to 19:00. The preschool arrival and departure time information for each child was documented by preschool teachers daily in the same in/out report that recorded children’s activity indoor or outdoor in 30 minutes periods from 7:00 to 19:00.”

Fig 1. Flowchart of participants.

Since the added figure of flowchart appears first in the manuscript, we have changed the numbering of other figures accordingly. 

3) While the authors use a statistical approach that allows for the nested data, it is unclear why teachers’ physical activity was aggregated at preschool-level (ultimately lowering total n and variability) and not clustered within classroom. Please explain the rationale behind not utilizing a 3-level approach – nesting students within teachers and teachers within schools. Do teachers interact with all children at the center? If not, please also include the average number of children per class.

Answer: Thank you for your comment and we apologize that we did not clarify the rationale behind the clustering. In Swedish preschools, there is no fixed classroom and all teachers take care of all preschoolers (Skolverket 2019). Although children can be divided into groups, the organization of children group varies between preschools and most children participate in different group constellations (Sheridan, Williams et al. 2014). Children can change groups and so can the teachers. Teachers do interact with all children by changing the children groups that they are responsible for or changing the children in their children groups. Therefore, we adopted a 2-level nesting, children-preschool, due to the organizational nature of Swedish preschools. 

We have clarified rationale for this 2-level nesting in the statistical analysis section of the manuscript (Page 10, line 234) as “There are no defined classrooms in Swedish preschools where all teachers take care and interact will all children in principle (Skolverket 2019). Although children can be divided into groups, these groups are not fixed and most children participate in different group constellations (Sheridan, Williams et al. 2014). Therefore, a 2-level nesting, children/teachers nested in preschools, was adopted in the LMM.”

References: 

Sheridan, S., P. Williams and I. Pramling Samuelsson (2014). "Group size and organisational conditions for children’s learning in preschool: a teacher perspective." Educational Research 56(4): 379-397.

Skolverket. (2019). "Curriculum for the Preschool Lpfö 18."

Minor comments:

1) In the introduction, please define preschool age (line 71, line 74). 1-5 years mentioned in into (line 78), yet study design is 3-5 years (line 115)

Answer: Thank you for your comment and we agree that two age range for preschoolers would be confusing for the readers. We have clarified why we have focused on 3-5 years old children in the method section (page 6, line 141) as “In Sweden, all children from the age of 1 to 5 are eligible to go to preschool. However, children aged 1-2 years are often separated in physical activity daily routines from children aged 3-5 years because of the difference in their development stage (Sheridan, Williams et al. 2014). Further, WHO have formulated different physical activity guidelines due to this variation in growth between 1-2 years old toddlers and 3-5 years old preschool children (WHO 2019). As such, preschool children of 3-5 years old were chosen as the study population of this research and all children between 3-5 years of age, at the participating preschools, were invited to participate.” 

References:

Sheridan, S., P. Williams and I. Pramling Samuelsson (2014). "Group size and organisational conditions for children’s learning in preschool: a teacher perspective." Educational Research 56(4): 379-397.

WHO (2019). WHO guidelines on physical activity, sedentary behaviour and sleep for children under 5 years of age. WHO. https://apps.who.int/iris/bitstream/handle/10665/311664/9789241550536-eng.pdf?sequence=1&isAllowed=y, WHO. ISBN 978-92-4-155053-6.

2) EPAO-SR is a large survey, which subscales/items of the EPAO-SR were used? (line 127)

Answer: Thank you for your comment, we agree that EPAO-SR is a big survey and our interest is in physical activity, therefore the nutrition part of the survey was not distributed and questions regarding preschool playground size and policy was especially the focus. We have clarified the use of EPAO-SR (page 7, line 159) as “The EPAO-SR instrument includes both questions about nutrition and physical activity and only questions regarding physical activity were distributed in this study. Subscales of EPAO-SR were used to measure environmental characteristics and formalized PA polices in the participating preschools. The specific questions asked were “How large is your preschool playground?” and “Does your preschool has written policy or any other written document about physical activity? Answers about playground size were categorized and modified to include the Swedish outdoor activity practice; (1) ≤200m2, (2) around 900m2, (3) >2700m2 and (4) outdoors activity (all time at the preschool is spent outdoors). Formalized PA policy was analyzed as a dichotomous variable (Yes/No), depending on if the preschool had any written policy concerning PA or not.”

3) Light PA (line 156) has been previously defined (line 73)

Answer: Thank you for your feedback to help us keep our paper concise. We have corrected Light PA into abbreviation (page 8, line 194) as “Steps were determined using the manufacturer’s step algorithm, using the normal filter. MVPA, LPA and ST were calculated based on cut‐offs and epochs developed specifically for the GT3X+ accelerometer, using Vm activity counts, in 4‐year‐old children” 

4) What is the cluster effect at the preschool-level, i.e. how much of the variance in the physical activity outcome data were attributed to school?

Answer: We appreciate your feedback and we concur that the cluster effect at preschool-level may be of interest for the readers and meta-analysis researchers with specific aim. Therefore, we have estimated the intra class correlation for each model used in the study and further presented these estimates in supplementary material S3 Table Preschool level cluster effect (Intra class correlation) in each Linear Mixed Model. As shown in the table, there was a small to modest ICC for all models and the ICC for LPA, steps and ST were somewhat higher than that of MVPA.

 Model 1 Model 2 

 ICC CI 95% ICC CI 95%

MVPA

Policy 0.078 0.028, 0.198 0.096 0.038, 0.220

Time outdoor 0.152 0.070, 0.298 0.130 0.056, 0.274

Playground size 0.128 0.048, 0.299 0.108 0.040, 0.264

LPA

Policy 0.308 0.183, 0.469 0.326 0.197, 0.488

Time outdoor 0.137 0.062, 0.276 0.139 0.063, 0.280

Playground size 0.286 0.160, 0.458 0.290 0.163, 0.461

Steps

Policy 0.369 0.235, 0.524 0.424 0.282, 0.579

Time outdoor 0.293 0.177, 0.445 0.306 0.186, 0.460

Playground size 0.348 0.208, 0.520 0.337 0.202, 0.505

ST

Policy 0.240 0.135, 0.390 0.238 0.132, 0.391

Time outdoor 0.245 0.140, 0.394 0.239 0.134, 0.390

Playground size 0.293 0.169, 0.460 0.279 0.157, 0.446

 Model 3 Model 4 

 ICC CI 95% ICC CI 95%

MVPA 0.112 0.040, 0.278 0.086 0.027, 0.024

LPA 0.099 0.036, 0.243 0.103 0.038, 0.250

Steps 0.264 0.146, 0.430 0.241 0.130, 0.423

ST 0.262 0.145, 0.427 0.247 0.133, 0.413

S3 Table Preschool-level cluster effect (Intra class correlation) in each Linear Mixed Model 

Model 1 = crude model each predictor independently, Model 2 = Model 1 adjusted for age, sex, BMI category

Model 3= all predictors jointly, Model4 = Model 3 adjusted for age, sex, age, BMI category

Abbreviations: MVPA = moderate to vigorous physical activity, 

LPA = light physical activity, ST = sedentary time

We have further clarified this in the revised manuscript. The numbering of other supplementary tables has also been changed in relation the additional new table with ICC estimates. 

Methods (page 10, line 238): “In addition, we estimated the intra class correlation for each mixed model to determine the preschool-level cluster effect.”

Results (page 15, line 330): “In sensitivity analysis, we estimated the intra class correlation of each model shown in S2 Table. We observed small to modest ICC for all models (and all outcomes); there was a small ICC for MVPA, and somewhat higher for Steps, LPA and ST (S3 Table). In addition, we ran all models included in S2 Table with additional adjustment made for parental education in a subset population of children (n=337) (S4 Table). S4 Table shows that none of the estimates are affected, to any substantial extent, by the introduction of parental education as a covariate. Finally, we ran descriptive analyses comparing children in the analytical sample (n=369) with children with incomplete accelerometer data or missing data on anthropometrics and or missing data on time spent in- and outdoors (n=35) (S5 Table). As shown in S5 Table, children excluded from analyses, due to missing data, had a higher prevalence of obesity (33% vs. 2%).”

5) Were their correlations between the independent variables? Are schools with larger playground areas more likely to spend more time outdoors? This may explain some of the findings for playground area for joint model.

Answer: Thank you for your questions and we agree that the relationship between the independent variables are complex. Although we observed an attenuation of playground size in our joint model which we argue is probable if time spent outdoors or PA policy mediates playground size relationship to PA, more complex relationships may exist. For instance, it is possible that these correlating factors interact, and such interactions may be of relevance for PA. However, due to our limited sample size and the cross-sectional design of our study we did not explore such complex interplay, and we suggest that future studies perform such detailed investigations

We have further discussed this in the manuscript (page 18, line 392) as “In the current study, we found a dose-response association between playground size and children’s levels of PA during preschool hours. However, this association was deteriorated when the association between playground size and PA was evaluated together with PA policy and time outdoors. Thus, the association between playground size and children’s PA may partly be explained by PA policy and time spent outdoors. Nevertheless, more complex relationships may exist. For example, it is possible that these correlating factors interact, and such interactions may be of relevance for PA. However, the potential complex interplay was not explored due to the limited sample size and the cross-sectional design of this study, future studies that perform such detailed investigations are warranted.”

6) Line 239 – unclear why only Fig 3/ steps is discussed as dose-response for independent models when MVPA and LPA show similar findings.

Answer: We apologize for the unclarity describing results about associations between preschool playground areas and preschool children’s PA, steps and ST. As you have mentioned the dose-response is similar in MVPA, LPA and steps in independent models, we concur with this and this is what we have described in the manuscript (unrevised manuscript line 239) as well: “the independent model shows dose-response association between preschool playground area and children’s PA and steps.” This observation was drawn from results shown in Fig1-3 rather than just Fig 3.

The wrong figure notation has caused this unclarity and we have now corrected this taking into consideration that we have added a new Fig 1 Flowchart of participants (page 14, line 269) as “As is shown in Figs 2-4, the independent model shows dose-response association between preschool playground area and children’s PA and steps. However, this dose-response association is not displayed when the preschool playground area is analyzed with other predictors simultaneously.”.

7) Results have no discussion of sedentary time findings.

Answer: We appreciate your comment about the sedentary time findings. However, the main goal of our study is to provide more evidence-base for intervention to promote MVPA rather than to decrease sedentary time. As is stated in the beginning of introduction (unrevised manuscript, page 3, line 66): “Total physical activity (PA), moderate to vigorous PA (MVPA) (Carson, Lee et al. 2017) and steps per day (Tudor-Locke, Craig et al. 2011) are positively associated with multiple health indicators in young children, while more conflicting findings have been reported for sedentary time (ST) (Cliff, Hesketh et al. 2016).” The benefits of MVPA is well-stablished in preschool children, while LPA is not as beneficial and the evidence about the relationship between sedentary time and health benefits is uncertain. In addition, there are a lot of developmental goals in the preschool age and some of them can be achieved while sedentary. For example, drawing develops fine object control and children’s appreciation of art - while often being a sedentary activity. We did not want to convey the message to preschool teachers that sedentary activity is “bad” and needs to be avoided which may limit their creativity in teaching and helping the children to achieve various developmental goals. However, by focusing on increasing MVPA, sedentary time would be decreased naturally assuming that MVPA would replace sedentary time. Therefore, we focused our discussion of findings on the potential to increase time in MVPA rather than to decrease time in sedentary behavior. 

References: 

Carson, V., E. Y. Lee, L. Hewitt, C. Jennings, S. Hunter, N. Kuzik, J. A. Stearns, S. P. Unrau, V. J. Poitras, C. Gray, K. B. Adamo, I. Janssen, A. D. Okely, J. C. Spence, B. W. Timmons, M. Sampson and M. S. Tremblay (2017). "Systematic review of the relationships between physical activity and health indicators in the early years (0-4 years)." BMC Public Health 17(Suppl 5): 854.

Cliff, D. P., K. D. Hesketh, S. A. Vella, T. Hinkley, M. D. Tsiros, N. D. Ridgers, A. Carver, J. Veitch, A. M. Parrish, L. L. Hardy, R. C. Plotnikoff, A. D. Okely, J. Salmon and D. R. Lubans (2016). "Objectively measured sedentary behaviour and health and development in children and adolescents: systematic review and meta-analysis." Obes Rev 17(4): 330-344.

Tudor-Locke, C., C. L. Craig, M. W. Beets, S. Belton, G. M. Cardon, S. Duncan, Y. Hatano, D. R. Lubans, T. S. Olds, A. Raustorp, D. A. Rowe, J. C. Spence, S. Tanaka and S. N. Blair (2011). "How Many Steps/Day are Enough? for Children and Adolescents." International Journal of Behavioral Nutrition and Physical Activity 8.

8) A limitation not noted is the dichotomous PA policy score. The EPAO-SR includes several policies that are specifically related to this study’s interest such as outdoors and adult-led activities. Reducing this to dichotomous variables may impact results as specific PA-related policies support meeting best practices at varying odds/prevalence (Dooley et al., 2020 Examining physical activity policies to practice implementation; Erinosho et al., 2016 Impact of policies on physical activity and screen time practices in 50 child-care centers in North Carolina; Sisson et al., 2012 Assessment of food, nutrition, and physical activity practices in Oklahoma child-care centers)

Answer: Thank you for your comment, and we concur that different policy have different impact on PA and to determine the specific policy content is a complex issue. We agree the specific policy content is important and preschool teachers did report what kind of policy they had in place in EPAO. 

The content of the of policy and number of preschools reported such policy is shown in the table below which has also been added as supplementary S6 Table. 

Formalized policy content Number of preschools reported such policy

Minimal daily time spent outdoors 7

Regular organized physical activity 5

Limited longer sedentary period 2

Teachers promote physical activity while being outdoors 6

Parents drop off/pick up children at preschool outdoors 7

Teachers participate in children’s active play 6

Teachers avoid using mobile phone/chatting with colleague while being outdoors 7

S6. Table Policy content in seven preschool reported formalized policy

While the content of policy was available and important, there was only 7/27 preschool that reported a formalized policy. We do not have enough power, nor did we expect to achieve high power while designing the study, to explore deeper into the content of policy. 

In addition, a policy is only effective if it is appropriately implemented; it may be inappropriate to make inference about the content of policy without further information about implementation and context (Howie and Stevick 2014). Therefore, we dichotomized the policy into yes/no and by analyzing it with other preschool modifiable factors, we strive to answer a different question with objective data: which of the modifiable factors would potential be most effective in increasing children PA during preschool time if addressed? 

It is an important question because it shed light on which factor to prioritize, given that resources for intervention are usually limited. However, we concur content of the policy plays an important role and this matter is further discussed (page 19, line 423) as “First, both preschool characteristics, assessed via the EPAO-SR, and the in- and outdoor reporting relied on preschool teacher’s self-reporting, which have several inherited biases. In addition, formalized PA policy was dichotomized into yes/no which may have disregarded the influence of policy’s specific content on PA outcomes (Erinosho, Hales et al. 2016, Dooley, Thi et al. 2020). Investigation further into content of formalized policy was hindered by the limited number of preschools that had formalized policy and unavailability of implementation information”.

References: 

Dooley, E. E., C. A. Thi, C. Browning, D. M. Hoelscher and C. E. Byrd-Williams (2020). "Examining physical activity policies to practice implementation: Results from the Texas Early Childhood Physical Activity Survey in non-Head Start childcare centers." Prev Med Rep 17: 101019.

Erinosho, T., D. Hales, A. Vaughn, S. Mazzucca and D. S. Ward (2016). "Impact of Policies on Physical Activity and Screen Time Practices in 50 Child-Care Centers in North Carolina." J Phys Act Health 13(1): 59-66.

Howie, E. K. and E. D. Stevick (2014). "The "ins" and "outs" of physical activity policy implementation: inadequate capacity, inappropriate outcome measures, and insufficient funds." J Sch Health 84(9): 581-585.

9) Would be helpful to include total accelerometer wear time and chi-square/t-test p-values in Table 1.

Answer: Thank you for your comment and we concur that more information on accelerometer wear time would help to understand the study sample as the aim of Table 1 is to provide an overview of the sample. 

However, we wish to argue that providing p-values in the table 1 is not necessary when presenting our descriptive data. We base this argument on two distinct notions, which are largely consistent with the recommendations from the STrengthening the Reporting of OBservational studies in Epidemiology (STROBE) guidelines. In fact, we believe that presenting p-values may misinform readers about the ‘relevance’ of factors.

First p-values could illustrate whether the difference we see between groups in Table 1 are probable under the null, but the differences manifested in table 1 can be confounded by unadjusted covariates and as such cannot be considered an unbiased estimate of the probability of observing said differences under the null. 

Secondly, our choice of confounders to be adjusted for in the adjusted models are based on casual diagrams rather than the significance of p-values in Table 1. Notably, informing covariate adjustment based on p-values could result in biased estimates as such cannot distinguish between mediators, confounders nor colliders on causal pathways. It is further noteworthy that the absence of a ‘statistically significant’ p-value may misinform covariate adjustment as covariates may be strongly associated to the outcome and/or become relevant confounders post-adjustment for other factors. In the unrevised manuscript we describe that we base our choice of confounders on a causal diagram (unrevised manuscript page 8, line 185) as “We analyzed each association between the exposures and outcomes independently and all predictors jointly in both unadjusted and adjusted models. Adjustments, in all models presented, were made for age of the child, sex and BMI (Dolinsky, Brouwer et al. 2011) that has been selected based on causal diagram (Williamson, Aitken et al. 2014).” 

Finally, to the best of our knowledge, PLOS adheres to the STROBE guidelines. The STROBE guidelines explicitly state that inferential measures should not be reported with descriptive characteristics: “Inferential measures such as standard errors and confidence intervals should not be used to describe the variability of characteristics, and significance tests should be avoided in descriptive tables. Also, P values are not an appropriate criterion for selecting which confounders to adjust for in analysis; even small differences in a confounder that has a strong effect on the outcome can be important.” - in Vandenbroucke et al. (2007), Strengthening the Reporting of Observational Studies in Epidemiology (STROBE): Explanation and Elaboration. PLOS Medicine (Vandenbroucke, von Elm et al. 2007)

However, we concur that providing accelerometer wear time is beneficial for understand the study sample and that it may inform comparability across studies. Since the outcomes in our study is PA level during preschool time. We have added the comparison of accelerometer wear time during preschool time and preschool time across the groups in the revised Table 1.

References:

Dolinsky, D. H., R. J. N. Brouwer, K. R. Evenson, A. M. Siega-Riz and T. Østbye (2011). "Correlates of sedentary time and physical activity among preschool-aged children." Preventing chronic disease 8(6): A131-A131.

Vandenbroucke, J. P., E. von Elm, D. G. Altman, P. C. Gøtzsche, C. D. Mulrow, S. J. Pocock, C. Poole, J. J. Schlesselman, M. Egger and S. I. for the (2007). "Strengthening the Reporting of Observational Studies in Epidemiology (STROBE): Explanation and Elaboration." PLOS Medicine 4(10): e297.

Williamson, E. J., Z. Aitken, J. Lawrie, S. C. Dharmage, J. A. Burgess and A. B. Forbes (2014). "Introduction to causal diagrams for confounder selection." Respirology 19(3): 303-311.

   

Table 1. Descriptive characteristics in relation to preschool level features

 Formalized PA Policy Playground area (m2) Time spent outdoors

 Total No Yes ≤200 Around 900 >2700 Out group Q1 Q2 Q3 Q4

 N= 369 N= 290 N= 79 N = 98 N= 69 N= 151 N= 51 N= 94 N= 93 N= 90 N= 92

Individual characteristics

 Boys, n (%) 204 (55.3) 125 (43.1) 40 (50.6) 55 (56.1) 34 (49.3) 88 (58.3) 27 (52.9) 40 (43) 50 (54) 38 (42) 37 (40)

 Age, mean (SD) 4.7 (0.1) 4.6 (0.8) 4.9 (0.7) 4.7 (0.7) 4.6 (0.9) 4.7 (0.7) 4.4 (0.9) 4.8 (0.8) 4.6 (0.8) 4.7 (0.7) 4.5 (0.8)

 BMI, mean (SD) 15.7 (0.1) 15.7 (2.8) 15.5 (2.1) 15.2 (3.8) 16.0 (1.2) 15.6 (2.5) 16.2 (1.7) 14.6 (4.3) 15.7 (2.0) 16.2 (1.1) 16.2 (1.6)

 Overweight, n (%) 25 (6.8) 20 (6.9) 5 (6.3) 7 (7.1) 6 (8.7) 8 (5.3) 4 (7.8) 5 (5) 6 (6) 8 (9) 6 (7)

 Obesity, n (%) 8 (2.2) 7 (2.4) 1 (1.3) 3 (3.1) 1 (1.4) 2 (1.3) 2 (3.9) 1 (1) 0 (0) 3 (3) 4 (4)

 Preschool children’s physical activity level during preschool time, mean (SD)

 MVPA (min) 39.2 (1.2) 37.2 (20.8) 46.4 (28.6) 33.5 (17.0) 36.1 (24.5) 43.3 (24.4) 42.0 (24.6) 37.4 (22.0) 33.5 (18.2) 42.3 (27.1) 43.7 (22.9)

 LPA (min) 258.8 (45.5) 258.5 (45.4) 259.8 (45.9) 240.3 (42.3) 247.6 (42.0) 266.9 (45.5) 285.4 (37.3) 226.7 (38.7) 248.6 (38.4) 273.2 (41.8) 287.7 (37.7)

 Steps (counts) 7343 (116) 7253 (2219) 7674 (2252) 6447 (1734) 6469 (1793) 7425 (1808) 10007 (2589) 6058 (1916) 6630 (1540) 7675 (1725) 9053 (2400)

 ST (min) 177.5 (2.4) 181.9 (45.2) 161.1 (44.0) 175.3 (45.7) 177.5 (36.4) 180.9 (53.5) 171.4 (29.4) 164.7 (42.0) 178.3 (42.5) 182.7 (50.7) 184.6 (45.3)

 Wear time (min) 446.1 (61.8) 448.6 (63.8) 437.1 (53.3) 419.9 (54.7) 431.2 (44.1) 462.2 (69.7) 469.0 (46.6) 401.5 (55.5) 431.2 (39.6) 467.6 (58.9) 485.8 (54.9)

Preschool time (min) 475.6 (62.1) 477.8 (64.2) 467.4 (53.6) 449.3 (54.5) 461.3 (45.2) 491.2 (70.1) 499.0 (46.6) 429.0 (53.7) 460.5 (40.1) 498.3 (58.8) 516.2 (54.8)

Parental characteristics

Education, n (%) N= 337 N= 265 N= 72 N= 87 N= 64 N= 140 N= 46 N= 81 N= 84 N= 88 N= 84

 University 271 (80.4) 214 (80.8) 57 (79.2) 73 (83.9) 52 (81.3) 106 (75.7) 40 (87.0) 70 (86) 66 (79) 66 (75) 69 (82)

Abbreviations: BMI = body mass index, MVPA = moderate to vigorous physical activity, LPA = light physical activity, ST = sedentary time, Q1-4 = quartile 1-4 

  

10) Would be helpful to include p-values for differences across groups for Table S4. Additionally, if possible, it would be beneficial to include the number of children excluded based on “incomplete accelerometer data”, “missing anthropometrics” and/or “missing data on time spent in- and outdoors” within the text for lines 276-278 or earlier in lines 196-197. Could there be center-level characteristics that led to exclusions – were excluded children random across centers or clustered in a few centers? Seems like children attending centers that do not have a formalized polices and smaller playground areas were more likely to be excluded.

Answer: Thank you for your comment and we agree that it is beneficial to provide information on missing values in further detail. We have answered your comments in three parts regarding a, the detailed exclusion procedure; b, the p values and number of missing values in Table S4; c, whether there is center level characteristics that led to exclusions.

a. Regarding the exclusion procedure

We understand the confusion caused by not specifying the number of children excluded at each exclusion step. One individual can have multiple missing value on different variables; therefore, we employed a stepwise exclusion strategy to clarify: First we made sure all data is valid by excluding all participants with less than 3 days or 10 hours/day of accelerometer data. Second, we excluded all participants with unrecorded preschool time - the factor later used to classify PA during preschool time. 

The exclusion procedure is illustrated by the new Fig 1 Flowchart of participants and is clarified in the revised manuscript (page 11, line 246) as “Fig 1 demonstrates the derivation of the analytical dataset. In total, 404 children and 92 preschool teachers from 27 preschools participated in the current study. First, 10 children and 6 teachers were excluded because they had less than 3 days or 10 hours/day of accelerometer data. Second, 25 children and 2 teachers were excluded due to missing recorded preschool hours, as such information were required to determine PA during preschool time. Thus, the final analytical sample comprised 369 children and 84 preschool teachers.”

Fig 1. Flowchart of participants

b. Regarding p values and number of missing values in S4 Table which is S5 Table in the revised manuscript

We strive to clarify the number of missing values in S5 Table, we have also added p-values in this table. Although the excluded participants seem to have statistically significant higher overweight/obesity and lower PA, it is important be bear in mind that this is based on limited observations due to the missing values that lead these participants to be excluded. Further, we wish to reemphasis points made regarding p-values in descriptive tables - they do not represent an unbiased estimate as they may be confounded. 

 Analytical dataset

(N=369) Excluded 

(Total N= 35) P value

Individual characteristics 

 Boys, n (%) (n=369) (n=35) 

 204 (55.3%) 18 (48%) 0.66

 Age, mean (SD) (n=369) (n=35) 

 4.7 (0.1) 4.6 (0.1) 0.90

…..Body measures (n=369) (n=25*) 

 BMI, mean (SD) 15.7 (0.1) 14.0 (1.4) 0.01

 Overweight, n (%) 25 (6.8%) 2 (7.4%) <0.001

 Obesity, n (%) 8 (2.2%) 9 (33.3%) 

School characteristics 

 Has formalized PA policy, n (%) (n=369) (n=35) 

 79 (27.2%) 3 (8.6%) 0.07

 Playground area (m2), n (%) (n=369) (n=35) 

 ≤200 98 (26.5%) 17 (48.6%) <0.001

 Around 900 69 (18.7%) 12 (34.3%) 

 >2700 151 (40.9%) 5 (14.3%) 

 Out group 51 (13.8%) 1 (2.9%) 

 Time spent outdoors (min), mean (SD) (n=369) (n=8**) 

 206.4 (5.6) 127.5 (36.2) 0.04

Child PA during preschool time, mean (SD) (n=369) (n=8**) 

 MVPA (min) 39.2 (1.2) 38.7 (9.6) 0.95

 LPA (min) 258.8 (45.5) 220.3 (51.5) 0.02

 Steps (counts) 7343 (116) 5775 (786) 0.05

 ST (min) 177.5 (2.4) 174.8 (18.7) 0.87

S5 Table. Comparison of descriptive characteristics between analytical dataset and excluded observations

PA = physical activity, BMI = body mass index, MVPA = moderate to vigorous physical activity, LPA = light physical activity, 

ST = sedentary time, Q1-4 = quartile 1-4

*Only 25 participants in the excluded datasets had available BMI data 

**Only 8 participants in the excluded datasets had available preschool time and time spent outdoors data 

c. Regarding whether there is center level characteristics that led to exclusions.

The exclusion is generally distributed across centers except that one preschool with no formalized policy and a small preschool playground accounted for 11 (31.4%) exclusions. The main exclusion was due to missing in/out report. This in/out report is a great challenge for the teachers to record whether the children is indoors or outdoors at every 30-minute-interval for every child during preschool time. Teachers usually divide responsibility to track children and report in a sub-form of in/out report before aggregating result. In this preschool with 11 missing data, the sub-report sheet for these 11 children were lost. Therefore, though it may seem like children from preschool with no formalized policy and a small garden are more likely to be excluded, but this exclusion was more likely due to accident that is unrelated to center-level characteristics. We made repeated attempts to retrieve and localize the lost report without success. 

We first clarified the distribution of excluded participations across preschools in sensitivity analysis (page 16, line 338) as “As shown in S5 Table, children excluded from analyses, due to missing data, had a higher prevalence of obesity (33% vs. 2%) and lower PA level. The excluded sample were generally distributed across the preschools except for that one preschool contributed to 11 (31.4%) exclusions due to loss of sub-report document for in/out report with information on preschool time and time spent outdoors. This preschool had the smallest preschool playground and did not have a formalized policy.”

We have further clarified the comparison between analytical dataset and excluded participants and the distribution of exclusion across preschools (page 19, line 429) as “Furthermore, our sensitivity analysis showed that children with incomplete data had a higher prevalence of obesity and lower PA level compared with those included in the main analyses. However, it is important to note that few observations (25 observations for obesity status and 8 observations for PA level) in the excluded sample due to missing value may limit the representativeness of this result in the excluded sample. Further, one preschool, with no formalized policy and a playground ≤ 200m2, contributed to 31.4% of the excluded participants due to loss of sub-document of in/out report with information on preschool time and time spent outdoors. This is of importance in relation to the center-level influence on missing data. Nevertheless, the constricted socioeconomic and body size distribution limit the generalizability of findings in the current study.”

Reviewer #2: Review of Manuscript for PLOS ONE

Thank you for the opportunity to review the paper entitled: ‘Preschool environment and preschool teacher’s physical activity and their association with children’s activity levels at preschool’. The manuscript provides detail of a study assessing the extent to which the preschool environment is associated with children’s physical activity, steps and sedentary time. This paper communicates original research and the findings provide some more insight into the activity levels of pre-schoolers with a novel inclusion of their teachers. I have reviewed the manuscript in its entirety. The paper is well-written and worthy of acceptance without revisions. There are two minor edits you may wish to attend to below. Congratulations.

Page 4 line 87- suggest changing the following wording: Studies on associations to Studies investigating associations

Page 15 line 311- add a word in the sentence: a review on the promotion of PA

Answer: Thank you very much for your revision and we are happy to hear that you have enjoyed our research paper. We have strived to provide more evidence regarding the association between modifiable factors in preschools and children’s PA in details. We would like to concur that this study provides novel insights into the understudied field of PA promotion in preschoolers with objective measure of PA on both preschool children and preschool teachers. As most 3-5 years old children attend preschools, regardless of the socioeconomic status of their parents, we believe that our research may contribute to potential strategies for environmental/organizational intervention in preschools that promote PA for all preschool children. 

We appreciate the minor edits you suggested, and we have made changes in the revised manuscript accordingly. 

(Page 5, line 110): “studies investigating associations between time spent outdoors at preschool and children’s levels of PA using objective measures on PA are also scarce. “

(Page 17, line 376): “However, a review on the promotion of PA in preschool children (Tremblay, Boudreau-Lariviere et al. 2012) highlights the importance of implementing policies concerning PA in preschools to promote children’s levels of PA during preschool hours.”

All references included in the point by point response to reviewers:

Berglind, D., L. Hansson, P. Tynelius and F. Rasmussen (2016). "Levels and Patterns of Objectively Measured Physical Activity and Sedentary Time in Four-Year-Old Swedish Children." J Phys Act Health: 1-23.

Berglind, D. and P. Tynelius (2017). "Objectively measured physical activity patterns, sedentary time and parent-reported screen-time across the day in four-year-old Swedish children." BMC Public Health 18(1): 69.

Boldemann, C., M. Blennow, H. Dal, F. Martensson, A. Raustorp, K. Yuen and U. Wester (2006). "Impact of preschool environment upon children's physical activity and sun exposure." Preventive Medicine 42(4): 301-308.

Bornstein, D. B., M. W. Beets, W. Byun and K. McIver (2011). "Accelerometer-derived physical activity levels of preschoolers: a meta-analysis." Journal of Science and Medicine in Sport 14(6): 504-511.

Broekhuizen, K., A.-M. Scholten and S. I. de Vries (2014). "The value of (pre)school playgrounds for children’s physical activity level: a systematic review." International Journal of Behavioral Nutrition and Physical Activity 11(1): 59.

Bronfenbrenner, U. (1979). The ecology of human development, Harvard university press.

Butte, N. F., W. W. Wong, J. S. Lee, A. L. Adolph, M. R. Puyau and I. F. Zakeri (2014). "Prediction of energy expenditure and physical activity in preschoolers." Med Sci Sports Exerc 46(6): 1216-1226.

Byrd-Williams, C. E., E. E. Dooley, C. A. Thi, C. Browning and D. M. Hoelscher (2019). "Physical activity, screen time, and outdoor learning environment practices and policy implementation: a cross sectional study of Texas child care centers." BMC Public Health 19(1): 274.

Carson, V., E. Y. Lee, L. Hewitt, C. Jennings, S. Hunter, N. Kuzik, J. A. Stearns, S. P. Unrau, V. J. Poitras, C. Gray, K. B. Adamo, I. Janssen, A. D. Okely, J. C. Spence, B. W. Timmons, M. Sampson and M. S. Tremblay (2017). "Systematic review of the relationships between physical activity and health indicators in the early years (0-4 years)." BMC Public Health 17(Suppl 5): 854.

Cliff, D. P., K. D. Hesketh, S. A. Vella, T. Hinkley, M. D. Tsiros, N. D. Ridgers, A. Carver, J. Veitch, A. M. Parrish, L. L. Hardy, R. C. Plotnikoff, A. D. Okely, J. Salmon and D. R. Lubans (2016). "Objectively measured sedentary behaviour and health and development in children and adolescents: systematic review and meta-analysis." Obes Rev 17(4): 330-344.

Dolinsky, D. H., R. J. N. Brouwer, K. R. Evenson, A. M. Siega-Riz and T. Østbye (2011). "Correlates of sedentary time and physical activity among preschool-aged children." Preventing chronic disease 8(6): A131-A131.

Dooley, E. E., C. A. Thi, C. Browning, D. M. Hoelscher and C. E. Byrd-Williams (2020). "Examining physical activity policies to practice implementation: Results from the Texas Early Childhood Physical Activity Survey in non-Head Start childcare centers." Prev Med Rep 17: 101019.

Dowda, M., W. H. Brown, K. L. McIver, K. A. Pfeiffer, J. R. O'Neill, C. L. Addy and R. R. Pate (2009). "Policies and characteristics of the preschool environment and physical activity of young children." Pediatrics 123(2): e261-266.

Erinosho, T., D. Hales, A. Vaughn, S. Mazzucca and D. S. Ward (2016). "Impact of Policies on Physical Activity and Screen Time Practices in 50 Child-Care Centers in North Carolina." J Phys Act Health 13(1): 59-66.

Finch, M., J. Jones, S. Yoong, J. Wiggers and L. Wolfenden (2016). "Effectiveness of centre-based childcare interventions in increasing child physical activity: a systematic review and meta-analysis for policymakers and practitioners." Obes Rev 17(5): 412-428.

Fossdal, T. S., K. Kippe, B. H. Handegard and P. Lagestad (2018). ""Oh oobe doo, I wanna be like you" associations between physical activity of preschool staff and preschool children." Plos One 13(11).

Gordon, E. S., P. Tucker, S. M. Burke and A. V. Carron (2013). "Effectiveness of physical activity interventions for preschoolers: a meta-analysis." Res Q Exerc Sport 84(3): 287-294.

Hibbing, P. R., D. R. Bassett and S. E. Crouter (2020). "Modifying Accelerometer Cut-Points Affects Criterion Validity in Simulated Free-Living for Adolescents and Adults." Res Q Exerc Sport: 1-11.

Hinkley, T., V. Carson and K. D. Hesketh (2015). "Physical environments, policies and practices for physical activity and screen-based sedentary behaviour among preschoolers within child care centres in Melbourne, Australia and Kingston, Canada." Child: Care, Health and Development 41(1): 132-138.

Hnatiuk, J. A., J. Salmon, T. Hinkley, A. D. Okely and S. Trost (2014). "A review of preschool children's physical activity and sedentary time using objective measures." Am J Prev Med 47(4): 487-497.

Howie, E. K. and E. D. Stevick (2014). "The "ins" and "outs" of physical activity policy implementation: inadequate capacity, inappropriate outcome measures, and insufficient funds." J Sch Health 84(9): 581-585.

Jones, R. A., T. Hinkley, A. D. Okely and J. Salmon (2013). "Tracking Physical Activity and Sedentary Behavior in Childhood: A Systematic Review." American Journal of Preventive Medicine 44(6): 651-658.

Marissa Hope, M., C. Ruth, M. Shannon and A. F.-L. Laura (2020). "Accelerometry Analysis Options Produce Large Differences in Lifestyle Physical Activity Measurement." Physiological Measurement.

Mehtälä, M. A. K., A. K. Sääkslahti, M. E. Inkinen and M. E. H. Poskiparta (2014). "A socio-ecological approach to physical activity interventions in childcare: a systematic review." International Journal of Behavioral Nutrition and Physical Activity 11(1): 22.

Migueles, J. H., C. Cadenas-Sanchez, U. Ekelund, C. Delisle Nystrom, J. Mora-Gonzalez, M. Lof, I. Labayen, J. R. Ruiz and F. B. Ortega (2017). "Accelerometer Data Collection and Processing Criteria to Assess Physical Activity and Other Outcomes: A Systematic Review and Practical Considerations." Sports Med.

Mikkelsen, B. E. (2011). "Associations between pedagogues attitudes, praxis and policy in relation to physical activity of children in kindergarten - results from a cross sectional study of health behaviour amongst Danish pre-school children." International Journal of Pediatric Obesity 6: 12-15.

Mueller, M. A.-O., R. Chimenti, S. Merkle and L. A.-O. Frey-Law "Accelerometry Analysis Options Produce Large Differences in Lifestyle Physical Activity Measurement. LID - 10.1088/1361-6579/ab94d4 [doi]." (1361-6579 (Electronic)).

Pearce, M., A. S. Page, T. P. Griffin and A. R. Cooper (2014). "Who children spend time with after school: associations with objectively recorded indoor and outdoor physical activity." Int J Behav Nutr Phys Act 11(1): 45.

Razak, L. A., S. L. Yoong, J. Wiggers, P. J. Morgan, J. Jones, M. Finch, R. Sutherland, C. Lecathelnais, K. Gillham, T. Clinton-McHarg and L. Wolfenden (2018). "Impact of scheduling multiple outdoor free-play periods in childcare on child moderate-to-vigorous physical activity: a cluster randomised trial." International Journal of Behavioral Nutrition and Physical Activity 15.

Sheridan, S., P. Williams and I. Pramling Samuelsson (2014). "Group size and organisational conditions for children’s learning in preschool: a teacher perspective." Educational Research 56(4): 379-397.

Skolverket. (2019). "Curriculum for the Preschool Lpfö 18."

Swerissen, H. and B. R. Crisp (2004). "The sustainability of health promotion interventions for different levels of social organization." Health promotion international 19(1): 123-130.

Tandon, P. S., B. E. Saelens, C. Zhou and D. A. Christakis (2018). "A Comparison of Preschoolers' Physical Activity Indoors versus Outdoors at Child Care." International Journal of Environmental Research and Public Health 15(11).

Tremblay, L., C. Boudreau-Lariviere and K. Cimon-Lambert (2012). "Promoting Physical Activity in Preschoolers: A Review of the Guidelines, Barriers, and Facilitators for Implementation of Policies and Practices." Canadian Psychology-Psychologie Canadienne 53(4): 280-290.

Tudor-Locke, C., C. L. Craig, M. W. Beets, S. Belton, G. M. Cardon, S. Duncan, Y. Hatano, D. R. Lubans, T. S. Olds, A. Raustorp, D. A. Rowe, J. C. Spence, S. Tanaka and S. N. Blair (2011). "How Many Steps/Day are Enough? for Children and Adolescents." International Journal of Behavioral Nutrition and Physical Activity 8.

Vandenbroucke, J. P., E. von Elm, D. G. Altman, P. C. Gøtzsche, C. D. Mulrow, S. J. Pocock, C. Poole, J. J. Schlesselman, M. Egger and S. I. for the (2007). "Strengthening the Reporting of Observational Studies in Epidemiology (STROBE): Explanation and Elaboration." PLOS Medicine 4(10): e297.

WHO (2019). WHO guidelines on physical activity, sedentary behaviour and sleep for children under 5 years of age. WHO. https://apps.who.int/iris/bitstream/handle/10665/311664/9789241550536-eng.pdf?sequence=1&isAllowed=y, WHO. ISBN 978-92-4-155053-6.

Williamson, E. J., Z. Aitken, J. Lawrie, S. C. Dharmage, J. A. Burgess and A. B. Forbes (2014). "Introduction to causal diagrams for confounder selection." Respirology 19(3): 303-311.

Attachment Submitted filename: Response to Reviewers.docx Click here for additional data file.

# Peer review: Decision Letter 1

**DOI:** 10.1371/journal.pone.0239838.r003

**Authors:** David Paul

15 Sep 2020

Preschool environment and preschool teacher’s physical activity and their association with children’s activity levels at preschool

PONE-D-20-01800R1

Dear Dr. Chen,

We’re pleased to inform you that your manuscript has been judged scientifically suitable for publication and will be formally accepted for publication once it meets all outstanding technical requirements.

Within one week, you’ll receive an e-mail detailing the required amendments. When these have been addressed, you’ll receive a formal acceptance letter and your manuscript will be scheduled for publication.

An invoice for payment will follow shortly after the formal acceptance. To ensure an efficient process, please log into Editorial Manager at http://www.editorialmanager.com/pone/, click the 'Update My Information' link at the top of the page, and double check that your user information is up-to-date. If you have any billing related questions, please contact our Author Billing department directly at authorbilling@plos.org.

If your institution or institutions have a press office, please notify them about your upcoming paper to help maximize its impact. If they’ll be preparing press materials, please inform our press team as soon as possible -- no later than 48 hours after receiving the formal acceptance. Your manuscript will remain under strict press embargo until 2 pm Eastern Time on the date of publication. For more information, please contact onepress@plos.org.

Kind regards,

David Paul, Ph.D.

Academic Editor

PLOS ONE

Additional Editor Comments (optional):

I congratulate the authors for completing revisions that appear to have addressed the concerns of the reviewers. I think the paper should be accepted.

Reviewers' comments:

Reviewer's Responses to Questions

**Comments to the Author**

1. If the authors have adequately addressed your comments raised in a previous round of review and you feel that this manuscript is now acceptable for publication, you may indicate that here to bypass the “Comments to the Author” section, enter your conflict of interest statement in the “Confidential to Editor” section, and submit your "Accept" recommendation.

Reviewer #1: All comments have been addressed

**********

2. Is the manuscript technically sound, and do the data support the conclusions?

The manuscript must describe a technically sound piece of scientific research with data that supports the conclusions. Experiments must have been conducted rigorously, with appropriate controls, replication, and sample sizes. The conclusions must be drawn appropriately based on the data presented. 

Reviewer #1: Yes

**********

3. Has the statistical analysis been performed appropriately and rigorously? 

Reviewer #1: Yes

**********

4. Have the authors made all data underlying the findings in their manuscript fully available?

The PLOS Data policy requires authors to make all data underlying the findings described in their manuscript fully available without restriction, with rare exception (please refer to the Data Availability Statement in the manuscript PDF file). The data should be provided as part of the manuscript or its supporting information, or deposited to a public repository. For example, in addition to summary statistics, the data points behind means, medians and variance measures should be available. If there are restrictions on publicly sharing data—e.g. participant privacy or use of data from a third party—those must be specified.

Reviewer #1: Yes

**********

5. Is the manuscript presented in an intelligible fashion and written in standard English?

PLOS ONE does not copyedit accepted manuscripts, so the language in submitted articles must be clear, correct, and unambiguous. Any typographical or grammatical errors should be corrected at revision, so please note any specific errors here.

Reviewer #1: Yes

**********

6. Review Comments to the Author

Please use the space provided to explain your answers to the questions above. You may also include additional comments for the author, including concerns about dual publication, research ethics, or publication ethics. (Please upload your review as an attachment if it exceeds 20,000 characters)

Reviewer #1: The authors did an excellent job addressing my comments. While the data are not fully available, the authors describe the legal and ethical restrictions.

**********

7. PLOS authors have the option to publish the peer review history of their article (what does this mean?). If published, this will include your full peer review and any attached files.

If you choose “no”, your identity will remain anonymous but your review may still be made public.

**Do you want your identity to be public for this peer review?** For information about this choice, including consent withdrawal, please see our Privacy Policy.

Reviewer #1: No

# Editor report: Acceptance letter

**DOI:** 10.1371/journal.pone.0239838.r004

**Authors:** David Paul

5 Oct 2020

PONE-D-20-01800R1 

Preschool environment and preschool teacher’s physical activity and their association with children’s activity levels at preschool 

Dear Dr. Chen:

I'm pleased to inform you that your manuscript has been deemed suitable for publication in PLOS ONE. Congratulations! Your manuscript is now with our production department. 

If your institution or institutions have a press office, please let them know about your upcoming paper now to help maximize its impact. If they'll be preparing press materials, please inform our press team within the next 48 hours. Your manuscript will remain under strict press embargo until 2 pm Eastern Time on the date of publication. For more information please contact onepress@plos.org.

If we can help with anything else, please email us at plosone@plos.org. 

Thank you for submitting your work to PLOS ONE and supporting open access. 

Kind regards, 

PLOS ONE Editorial Office Staff

on behalf of

Dr. David Paul 

Academic Editor

PLOS ONE
